# Temporal modulation of gene expression in a controlled *Schistosoma mansoni* human infection model

**DOI:** 10.3389/fimmu.2025.1707749

**Published:** 2025-12-16

**Authors:** Dawei Li, Chuan Jiang, Wenyu Zhu, Emma L. Houlder, Koen A. Stam, Jan Pieter R. Koopman, Marijke Langenberg, Ranmali Kavishna, Sara Torkzadeh, Björn Andersson, Josefine Persson, Aravindan Kalyanasundaram, Mumtaz Y. Balkhi, Desalegn W. Kifle, Aryandra Arya, Hyon Jin Jeon, Birkneh T. Tadesse, Sean A. Gray, Darrick Carter, Alison M. Elliott, Florian Marks, Ali M. Harandi, Meta Roestenberg, Afzal A. Siddiqui

**Affiliations:** 1 Department of Immunology & Molecular Microbiology, Center for Tropical Medicine and Infectious Diseases, Texas Tech University Health Sciences Center, Lubbock, TX, United States; 2 Leiden University Center for Infectious Diseases, Leiden University Medical Center, Leiden, Netherlands; 3 Department of Microbiology & Immunology, Institute of Biomedicine, Gothenburg, Sweden; 4 Bioinformatics Core Facility, Sahlgrenska Academy, University of Gothenburg, Gothenburg, Sweden; 5 International Vaccine Institute, Seoul, Republic of Korea; 6 Cambridge Institute of Therapeutic Immunology and Infectious Disease, University of Cambridge School of Clinical Medicine, Cambridge, United Kingdom; 7 Madagascar Institute for Vaccine Research, University of Antananarivo, Antananarivo, Madagascar; 8 PAI Life Sciences Inc., Seattle, WA, United States; 9 Medical Research Council, Uganda Virus Research Institute and London School of Hygiene & Tropical Medicine Uganda Research Unit, Entebbe, Uganda; 10 Heidelberg Institute of Global Health, University of Heidelberg, Heidelberg, Germany; 11 Vaccine Evaluation Center, BC Children’s Hospital Research Institute, University of British Columbia, Vancouver, BC, Canada

**Keywords:** controlled human infection, parasite, RNA-Seq, schistosomiasis (*S. mansoni*), single-sex

## Abstract

**Background:**

Schistosomiasis is caused by parasitic blood flukes of the genus *Schistosoma*. Despite ongoing mass drug administration efforts, the disease remains a major public health burden in endemic regions. A better understanding of early host responses to schistosomiasis is critical for developing effective vaccines and therapeutics.

**Methods:**

We conducted a longitudinal transcriptomic study of peripheral blood samples from 30 *Schistosoma*-naïve volunteers participating in two controlled human infection trials with male- or female-only *S. mansoni* cercariae. Blood was collected at six time points over 20 weeks post-infection. Whole-transcriptome RNA sequencing and integrative analyses, including differential gene expression, gene set enrichment, protein interaction networks, co-expression clustering, and immune module profiling, were employed to characterize temporal modulation of genes related to immune responses.

**Results:**

Robust and highly time-dependent transcriptional responses were observed, peaking at Week 4 post-infection. Differential gene expression and pathway analyses revealed activation of immune responses, including type I and II interferon signaling, chemokine-mediated pathways, and antigen presentation. Notably, both Th1 and Th2 signatures were evident at Week 4. Key immune hubs included *IFNG*, *TNF*, and *IL1B*, along with transcriptional regulators such as *STAT1* and *IRF7*. Blood transcription module analysis further highlighted transient activation of interferon and plasma cell-related responses.

**Conclusions:**

This study provides a comprehensive transcriptional map of early host responses to *S. mansoni* infection in humans. The findings underscore the central role of interferon pathways, early mixed Th1/Th2 polarization, and inflammation-associated gene signatures in shaping host response to *S. mansoni* infection. These insights may inform the rational design of vaccines and biomarkers for schistosomiasis.

## 1 Introduction

Schistosomiasis, also known as bilharzia, is a neglected tropical disease caused by trematode blood flukes of the genus, *Schistosoma*. The disease disproportionately affects low-income populations across 78 countries worldwide (1). Among the six *Schistosoma* species known to infect humans, *S. mansoni* and *S. haematobium* are the most prevalent and account for the majority of schistosomiasis-associated morbidity and mortality (2, 3). Morbidity primarily results from the deposition of eggs, rather than adult worms, which become trapped in host tissues such as the liver, intestines, or urinary bladder (4, 5). These matured eggs elicit macrophage reactions and secretion of lytic enzymes leading to granuloma formation. Granulomas induce prolonged chronic inflammation resulting in tissue fibrosis. An adaptive T helper 2 (Th 2) response is also generated against egg antigens. In a chronic disease, egg deposition in various organs results in hepatosplenomegaly for intestinal/hepatic schistosomiasis and eggs settling in urinary bladder cause urogenital disease (4–7). According to the World Health Organization (WHO), approximately 240 million people are infected with schistosomiasis, with nearly 91% of cases occurring in Africa. Globally, an estimated 800 million people are at risk of infection (3).

Despite substantial efforts to eliminate schistosomiasis, including snail control, community education, and mass drug administration (MDA) with praziquantel (PZQ), the disease remains a major public health challenge and continues to cause significant morbidity in endemic countries (2, 6). While PZQ is effective against adult worms of *Schistosoma* spp (4, 6, 8), MDA has several limitations. These include the need for repeated administration -which is difficult especially in hard-to-reach areas in countries endemic for schistosomiasis, rapid reinfection, and its limited efficacy against eggs and immature worms. Furthermore, concerns about potential drug resistance underscore the urgent need for alternative control strategies, including vaccines (9–13).

Developing successful interventions against schistosomiasis will require a systems-level understanding of host-pathogen interactions. As part of our ongoing efforts to support human schistosomiasis vaccine development, we have established safe, single-sex (male-only or female-only) controlled human infection (CHI) models in *Schistosoma*-naïve volunteers (14–17). While these CHI models differ from natural infections in endemic settings, they have greatly enhanced our understanding of host responses during the acute phase of *Schistosoma* infection.

CHI models provide experimental flexibility to selectively contain and investigate single sex (male or female only) *Schistosoma* infection. In contrast, natural infection cannot be controlled where infection can occur from copulated parasite or multiple infections as well as re-infections. Furthermore, endemic population compared to CHI models presents with previous exposure history complicating the elucidation of worm mediated true immunological response. Moreover, CHI models offer a valuable platform for early evaluation of vaccine candidates, allowing for better candidate selection, reduced failure rates, and more efficient progression to large-scale clinical trials (18).

In this report, we have analyzed samples from two CHI trials by performing whole-transcriptome Illumina sequencing of peripheral blood collected from 30 participants at six time points over a 20-week period following *S. mansoni* cercarial challenge. Our differential gene expression and pathway analyses revealed significant, time-dependent changes in host gene expression. To our knowledge, this is the first study to characterize immunological and molecular responses to *S. mansoni* in humans using CHI model of *Schistosoma* infection in non-endemic population, providing novel insights into host-pathogen interactions during the initial stages of infection. However, transcriptomic analysis of peripheral blood samples collected from naturally *S. mansoni*-infected children in endemic Albert Nile region of Uganda, genes associated with fibrosis have been identified (19).

## 2 Materials and methods

### 2.1 Subject recruitment

The two controlled human *Schistosoma* infection dose-escalation trials were approved by the Institutional Medical Ethical Research Committee of Leiden University Medical Center (Institutional Review Board P16.111 and P20.015), in accordance with the European Clinical Trial Directive 2001/20/EC, ICH-GCP guidelines, and the Declaration of Helsinki. Written informed consent was obtained from all participants. A total of 30 volunteers (designated S1 to S30) were successfully recruited. In the first CHI trial (NCT02755324), 17 volunteers (6 male and 11 female adult volunteers) were exposed with either 10 (n = 3), 20 (n = 11), or 30 (n = 3) male *S. mansoni* cercariae. The study groups were small (3–11 volunteers), showing differences in age (median 20, 23 and 30) and -man/woman ratio (0%, 33%, and 45% men). In the second CHI trial (NCT04269915), 13 volunteers (5 male and 8 female adult volunteers) were challenged with 10 (n = 3) or 20 (n = 10) female *S. mansoni* cercariae. Further details on recruitment are available in previous publications (16, 17). Participants exposed with male cercariae received PZQ at Week 12, while those exposed with female cercariae were treated at both Week 8 and Week 12.

### 2.2 Blood collection and RNA extraction

Total RNA was extracted and purified from whole blood collected in PAXgene^®^ Blood RNA tubes from 30 participants at six time points, including baseline (prior to exposure, Week 0) and every four weeks thereafter until Week 20 post-exposure (*i.e*., Weeks 0, 4, 8, 12, 16, and 20; details in **Supplementary Figure S1**), using the Total RNA extraction kit (RNeasy kit, Qiagen). RNA quantity and quality were assessed using the NanoDrop 2000 (Thermo Fisher Scientific) and the 2200 TapeStation Automated Electrophoresis System (Agilent Technologies). Hemoglobin mRNA depletion was performed for each sample. Samples with RNA Integrity Number greater than 6.0 were selected for sequencing.

### 2.3 Whole-transcriptome sequencing

The Ribo-Zero Gold kit and TruSeq Stranded Total RNA Sample Preparation Kit (Illumina, Inc., San Diego, CA) were used to deplete rRNA from total RNA and to prepare stranded RNA libraries. Paired-end whole-transcriptome sequencing (100 base pairs) was performed on the NovaSeq 6000 platform (Illumina, Inc., San Diego, CA) at the Clinical Genomics Core Facility in Gothenburg, Sweden.

### 2.4 Summary of RNA-Seq data analyses

A comprehensive transcriptomic analysis pipeline was implemented (**Supplementary Figure S2**), beginning with stringent quality control of raw FASTQ sequencing reads using FastQC, Fastp, SortMeRNA, and Kraken2/Bracken to remove low-quality sequences and contaminants. Cleaned reads were aligned to the CHM13 human reference genome using STAR, and gene expression was quantified with *featureCounts* (20), followed by normalization using *edgeR* (21). Principal component analysis (PCA) was performed to identify outliers, and differential gene expression analysis was conducted using *edgeR* across various biological variables. Functional interpretation of gene expression data was carried out using Gene Ontology (GO) enrichment analysis on differentially expressed genes (DEGs) and Gene Set Enrichment Analysis (GSEA) on the entire ranked gene list, both implemented via the *clusterProfiler* package. Immune-related GO terms were further identified via keyword matching. Time-resolved gene expression patterns were explored using Fuzzy C-means clustering, with meta-clustering to identify consistent co-expression groups, followed by GO and transcription factor enrichment analyses. Weighted Gene Co-expression Network Analysis (WGCNA) was used to identify gene modules associated with experimental conditions. Finally, individual-level transcriptional responses were assessed using the BloodGen3Module framework combined with GSEA to reveal functionally enriched gene modules over time. Genes were considered significant if the fold change was > 1.5 (|log_2_FC| > 0.58) and FDR-adjusted P value (*P*_adj_) was < 0.05, and GO terms were considered significant if *P*_adj_ was < 0.05. Based on previous studies (22, 23), moderate differential expression thresholds (|log_2_FC| > 0.58, *P*_adj_ < 0.05) were used to balance the need to classify biologically relevant changes among the different variables of interest (such as before and after infection) while upholding statistical power. Consistently, the changes in gene expression obtained in this study appeared to resemble early stages of *S. mansoni* infections under natural conditions. The detailed methods are provided in the **Supplementary Methods**.

## 3 Results

### 3.1 Research samples

**Figure 1:**
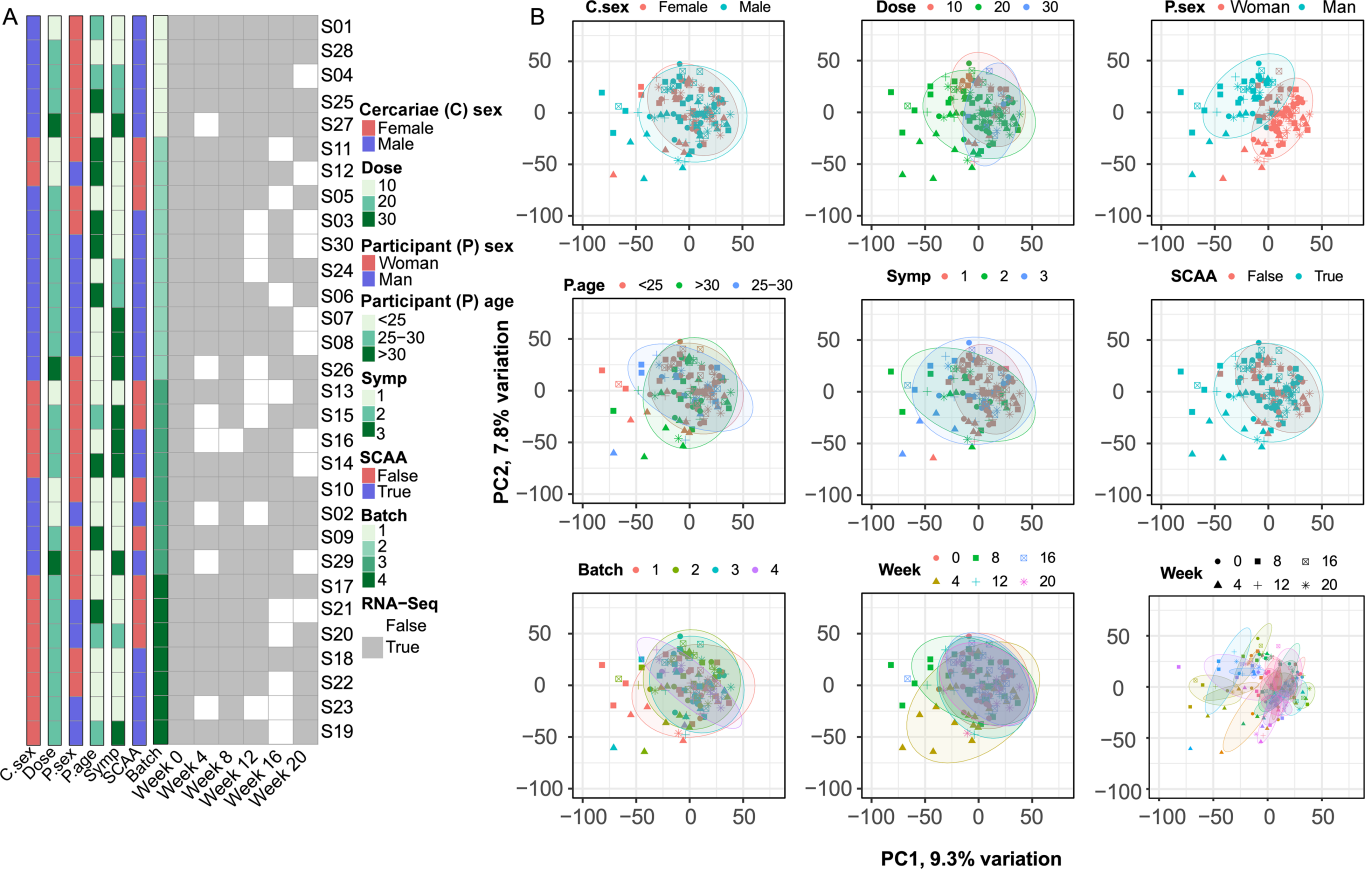
Overview of study participants and sampling and principal component analysis. **(A)** Overview of study participants and sampling. **(B)** PCA of RNA-Seq samples by experimental variables. Each dot represents an individual sample, colored by subgroup. Clustering is shown for variables including cercariae sex (C. sex), dose (Dose), participant sex (P. sex), age (P. age), symptom grade (Symp), circulating anodic antigen (SCAA), sequencing batch, and time point.

Each participant was sampled at six time points: before exposure to cercariae (baseline, Week 0) and at Weeks 4, 8, 12, 16, and 20 post exposure. Some of the participants did not came back for every follow-up/scheduled visit in some cases. Therefore, samples for all of the time points are not complete. Thus, the total number of samples collected was 145, however, one timepoint was excluded based on quality control criteria. **Figure 1A** summarizes the 144 RNA-Seq samples collected from all 30 participants, broken down by experimental variables such as parasite sex and dose, participant sex and age, symptom grade (defined in our previous publication) (16, 17), worm-excreted circulating anodic antigen in serum (SCAA status-SCAA was applied to determine the presence and degree of infection; SCAA was considered positive if at least one value ≥ 1.0 pg/ml was detected before Week 8, otherwise, it was considered negative), and sequencing batch. A detailed breakdown of sample sizes across all subgroups is provided in **Supplementary Table 1**.

Several significant associations were identified: A strong association (*P_adj_* = 3 × 10^-5^) between parasite sex and SCAA was observed, with 80% of samples from participants exposed to male cercariae testing SCAA-positive, compared to only 44% in the female exposure group (**Supplementary Table 1B**). Additionally, cercariae dose was positively correlated with symptom severity (*P_adj =_* 10 × 10^-13^) and SCAA positivity (*P_adj_* = 4 × 10^-5^). Samples exposed to the highest dose (30 cercariae) showed severe symptoms, while all samples from the lowest dose group (10 cercariae) showed no symptoms (**Supplementary Table 1C**). Furthermore, all samples from the 30-cercariae group were SCAA positive, whereas only 33% of the 10-cercariae group were SCAA positive (**Supplementary Table 1C**). A positive association (*P_adj_* = 3 × 10^-5^) between symptom severity and SCAA positivity was also observed. Specifically, 90% of samples from participants with severe symptoms were SCAA positive, while only 48% of samples from participants with no symptoms were SCAA positive (**Supplementary Table 1F**).

### 3.2 RNA-Seq data quality

The strandedness of sequencing reads was inferred to be reverse, consistent with the actual library preparation strategy, as expected. Three samples were sequenced twice, and their FASTQ files were merged for analysis. On average, each sample generated approximately 40 million reads. The GC content of these reads ranged from 47% to 57%. The total mapping percentage for all samples was greater than 90%, with 75% of reads uniquely mapped. Of the uniquely mapped reads, 16% were splice reads, with over 80% corresponding to known splicing junctions, approximately 8% to novel junctions, and the remainder to partial novel junctions. Additionally, 5% of the total mapped read pairs were mapped away from each other, suggesting structural variations. Of the 145 samples, one sample (S16 at Week 12) exhibited an excessive percentage (81.7%) of rRNAs and was therefore discarded. The remaining samples had either very low rRNA levels or levels that did not affect the analysis. Kraken2 and Bracken results indicated no significant contamination in the data. These analyses resulted in a total of 144 samples available for subsequent differential gene expression analysis. **Supplementary Figure S3** displays the numbers of reads, percentages of aligned reads, uniquely aligned reads, rRNA content, and percentage of human reads for each sample.

### 3.3 Clustering analysis of samples

PCA of 144 RNA-Seq samples indicated that no distinct clustering was observed based on sequencing batches (**Figure 1B**). Across the six time points, samples from Weeks 4 and 8 deviated from baseline (Week 0) and other time points. Another important observation from the PCA was a clear separation between man and woman participants, indicating that expected sex-related differences were captured by the transcriptome profiles. Additionally, samples from different time points within the same participant tended to cluster closely together, as shown in both PCA plots and pairwise sample correlation heatmaps (**Supplementary Figure S4**). Therefore, participant identifiers were used as covariates in subsequent differential gene expression analyses of time points to account for potential biases related to individual participants, including sex.

### 3.4 Differential gene expression analyses

**Figure 2:**
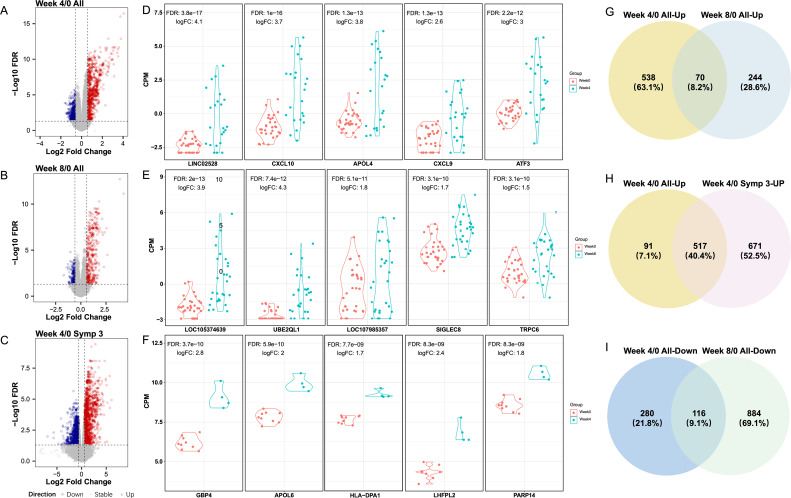
Differential gene expression analysis. **(A-C)** Differentially expressed genes by comparing Week 4 and Week 8 to Week 0, including valcono plots of DEGs for comparisons Week 4 vs. 0 and Week 8 vs. 0 in all samples **(A, B)**, and Week 4 vs. 0 in the Symp:3 subgroup **(D-F)**. The top five upregulated DEGs identified at **(D)** Week 4/0 (all samples), **(E)** Week 8/0 (all samples), and **(F)** subgroup Week 4/0 Symp:3. Venn diagrams comparing upregulated DEGs: between **(G)** Weeks 4/0 (all samples) and **(H)** Weeks 8/0 (all samples); **(I)** between Weeks 4/0 (all samples) and Weeks 4/0 (Symp:3).

Overall, differential gene expression analyses identified a substantial number of DEGs across various comparisons (**Supplementary Figures S5C, D**) show the number of DEGs by different time points with Week 0; and the full list is provided in **Supplementary Table 2**. As all key experimental variables were evenly distributed across time points (**Supplementary Table 1A**), a time-series analysis using all samples was conducted by comparing to the baseline (Week 0). Volcano plots for the comparisons between Week 4 and Week 0 (Week 4/0) and between Week 8 and Week 0 (Week 8/0) are shown in **Figures 2A, B**. When compared with baseline (Week 0) at all sample level, the Week 4/0 comparison yielded the highest number of DEGs (608 upregulated, 396 downregulated), followed by Week 8/0 (314 upregulated, 141 downregulated), with 142 DEGs (70 upregulated (**Figure 1G**) and 72 downregulated) showing significant expressions at both time points. No DEGs were detected after Week 8 relative to baseline, consistent with the timing of PZQ administration after Week 8 and/or Week 12. The top 10 upregulated genes at Week 4 were *LINC02528* (log_2_ fold change or log_2_FC = 4.1, *P_adj_* = 4 × 10^-17^), *CXCL10*, *APOL4*, *CXCL9*, *ATF3*, *CFB*, *LOC112268418*, *ACOD1*, *GBP4*, and *FAM225A*, while the top 10 downregulated ones were *LOC122526780* (log_2_FC = -0.7, *P_adj_*= 2× 10^-6^), *FAM153A*, *AOC2*, *KAZN*, *IRS2*, *LOC101927759*, *WDR86*, *DLEC1*, *BAIAP3*, and *ITGB4*. The top 10 upregulated genes at Week 8 were *LOC105374639* (log_2_FC = 3.9*, P_adj_* = 2 × 10^-12^), *UBE2QL1*, *LOC107985357*, *SIGLEC8*, *TRPC6*, *PTGDR2*, *PLAAT5*, *ZBTB42*, *CYP7B1*, and *ADORA3*, while the top 10 downregulated ones were *LOC124901656* (log_2_FC = -1.0, *P_adj_* = 1.6 × 10^-5^), *ARHGEF40*, *RAB36*, *LOC124903207*, *LINC02903207*, *LINC0975*, *LOC107985769*, *LOC102724560*, *AATK*, *MANSC1*, and *THBD*. The top five upregulated DEGs at Week 4 and Week 8 compared to Week 0 are shown in **Figures 2D, E**. By comparing DEGs between Week 8 and Week 4, we observed that many of the top upregulated genes at Week 4 became downregulated by Week 8. Nevertheless, numerous genes remained upregulated at Week 8 compared to baseline (**Supplementary Figure S11A**). These changes highlight that exposure to *S. mansoni* cercariae induces significant and time-dependent transcriptional changes in human hosts, with hundreds to thousands of genes being either upregulated or downregulated at different time points post challenge.

Differential gene expression analyses were also performed in subgroups stratified by experimental variables such as cercariae sex (C.sex), dose (Dose), participant sex (P.sex), age (P.age), symptom grade (Symp), and SCAA status (SCAA) (**Supplementary Figures S5C, D**; the full list provided in **Supplementary Table 2**). Many DEGs were found at Week 4 and Week 8 compared to the baseline. The numbers of DEGs detected in these subgroups were comparable to those observed in all participants as long as the subgroups had sample sizes larger than 10 (**Supplementary Figures S5A, B**). Although fewer DEGs were identified in subgroups with smaller sample sizes (e.g., Symp:1, P.age:25-30, and Dose:10), their fold-change patterns were similar to those observed in the corresponding larger groups as shown in volcano plots (**Supplementary Figures S6, 7**).

The subgroup of man only participants (n = 11) did not yield any detectable DEG across all six time points. On the other hand, more DEGs were identified in the subgroup with severe symptom grade (Symp:3, n = 9). Although only four participants from this subgroup were sampled at Week 4, a total of 1,188 upregulated and 1,000 downregulated genes were identified. Compared to the DEGs identified in all participants, approximately half (671) of the upregulated DEGs were unique to this subgroup, while the remaining (517) were also found in all participants (**Figure 2G**). For downregulated genes, 884 were unique to subgroup Symp:3, while the rest 116 were also identified in all participants (**Figure 2I**). The top 10 upregulated genes in Symp:3 were: *GBP4* (log_2_FC = 2.8, *P_adj_* = 4 × 10^-10^), *APOL6*, *HLA-DPA1*, *PARP14*, *LHFPL2*, *GBP5*, *LAP3*, *STAT1*, *PDCD1LG2*, *APOL1*, while the top 10 downregulated genes were *LINC00926* (log_2_FC = -1.7, *P_adj_* = 2 × 10^-6^)*, BCAS4, RUBCNL, NT5E, ABCC5, CBX7, COL4A3, ZNF860, GDF7*, and *RIC3*. The top five upregulated genes are shown in **Figure 2F**.

When examining the intersection of DEGs across various subgroups, some upregulated genes overlapped among subgroups, while most downregulated genes tended to be unique to specific subgroups (**Supplementary Figures S11B, C**). Moreover, the ranges of log_2_ fold changes for upregulated genes were more pronounced than those for downregulated genes. Thus, the upregulated DEGs are likely to play more primary roles in the response to *S. mansoni* exposure. In contrast, the downregulated DEGs may represent complementary changes that help facilitate activation of relevant pathways (24). Additionally, the Symp:3 subgroup at Week 4 exhibited the highest numbers of both upregulated and downregulated unique DEGs, suggesting that unique pathways are possibly induced in participants with the most severe symptoms after challenge.

### 3.5 Functional enrichment analysis post *S. mansoni* cercariae challenge

To understand changes of the key biological processes caused by the *S. mansoni* cercariae challenge, three enrichment analysis methods, GO enrichment analysis, KEGG and GSEA, were performed. Under certain conditions, pathways may be affected by coordinated but modest changes in gene expression, even when these individual genes are not classified as DEGs. Compared to DEG-based analysis which emphasizes genes with large expression changes, GSEA can capture more subtle, coordinated shifts across entire gene sets. Therefore, not surprisingly, more GO terms were enriched by GSEA. At all sample level, 307 GO terms upregulated and 14 downregulated at Week 4 compared to Week 0 (**Supplementary Figures S5G, H**, the full list in **Supplementary Table 4A**). On the contrary, for the same comparison, GO enrichment analysis only identified 164 upregulated and 10 downregulated GO terms (**Supplementary Figures S5E, F**; full list in **Supplementary Table 3**), while KEGG obtained 129 upregulated terms and no downregulated one (**Supplementary Figures S5I, G**; full list in **Supplementary Table 4B**). Moreover, a significant number of GO terms were enriched at later time points (Weeks 12, 16, and 20) by GSEA, while few or even no terms were identified at these later weeks when using the other two methods due to the absence of DEGs. Although the three methods vary from each other, the results obtained were consistent. The two main findings were: first, the identified GO terms (pathways in KEGG) were highly time dependent, and Week 4 exhibited the highest number of enriched GO terms compared to baseline (Week 0); second, many immune related GO terms were identified suggesting that many immune responses were probably activated after *S. mansoni* cercariae challenge. In our study, GSEA was selected for the following analysis and discussion and the results obtained by GO enrichment analysis and KEGG were not further discussed.

The most upregulated GO term at Week 4 was GO:0051607 (defense response to virus, NES = 2.96, *P_adj_* = 4 × 10^-9^) in all samples, followed by GO:0009615 (response to virus, NES = 2.89, *P_adj_* = 4 × 10^-9^). Many GO terms related to various immune responses were significantly upregulated at Week 4 in all samples, such as responses to viruses (e.g., GO:0051607, GO:0009615), regulation and response to interferons (e.g., GO:0071357, GO:0140888), responses of adaptive (e.g., GO:0002460, GO:0002822) and innate (e.g., GO:0002758, GO:0002227) immune responses, regulation and response to cytokines (e.g., GO:0001819, GO:0002367), regulation of T cell activation, differentiation, and proliferation (e.g., GO:002709, GO:0050868, GO:0042098), and processing and presentation of antigen (e.g, GO:0002478, GO:0002504). Meanwhile, many GO terms related to various aspects of cell cycle were strongly upregulated at Week 4 when compared to Week 0, such as those involved in cell division (e.g., GO:0000280, GO:0007059, GO:005000, GO:0007094) and mitotic cell cycle (e.g., GO:0007094, GO:0071174, GO:0065004, GO:0044839). Such changes suggest that *S. mansoni* cercariae challenge might alter host’s cell cycle progression at the earlier stage. At Week 8, only three GO terms were significantly upregulated including two associated with response to virus (GO:0051607, GO:0009615), followed by GO term GO:0002460 (adaptive immune response based on somatic recombination of immune receptors built from immunoglobulin superfamily domains).

A similar trend was observed in subgroup analyses, with more GO terms enriched from upregulated DEGs, predominantly at Week 4 (**Supplementary Figures S5E, F**). Some subgroups stood out with significantly more enriched GO terms. For example, SCAA: True, Symp: 3, P.age: 25, and P. sex: Women participants showed higher numbers of upregulated GO terms at Week 4 compared to their respective counterparts at Week 0. Symp:3, which had the highest number of upregulated DEGs, also exhibited the largest number of enriched GO terms (330 upregulated), many of which were immune-related (**Supplementary Table 4A**). In contrast, despite 1,000 downregulated DEGs in Symp:3 at Week 4, only nine GO terms were identified. Similarly, other subgroups yielded very few enriched GO terms from downregulated DEGs at Week 4. These observations support our hypothesis that downregulated genes may reflect compensatory responses to earlier pathway activation, resulting in fewer or more diffuse GO term enrichments.

**Figure 3:**
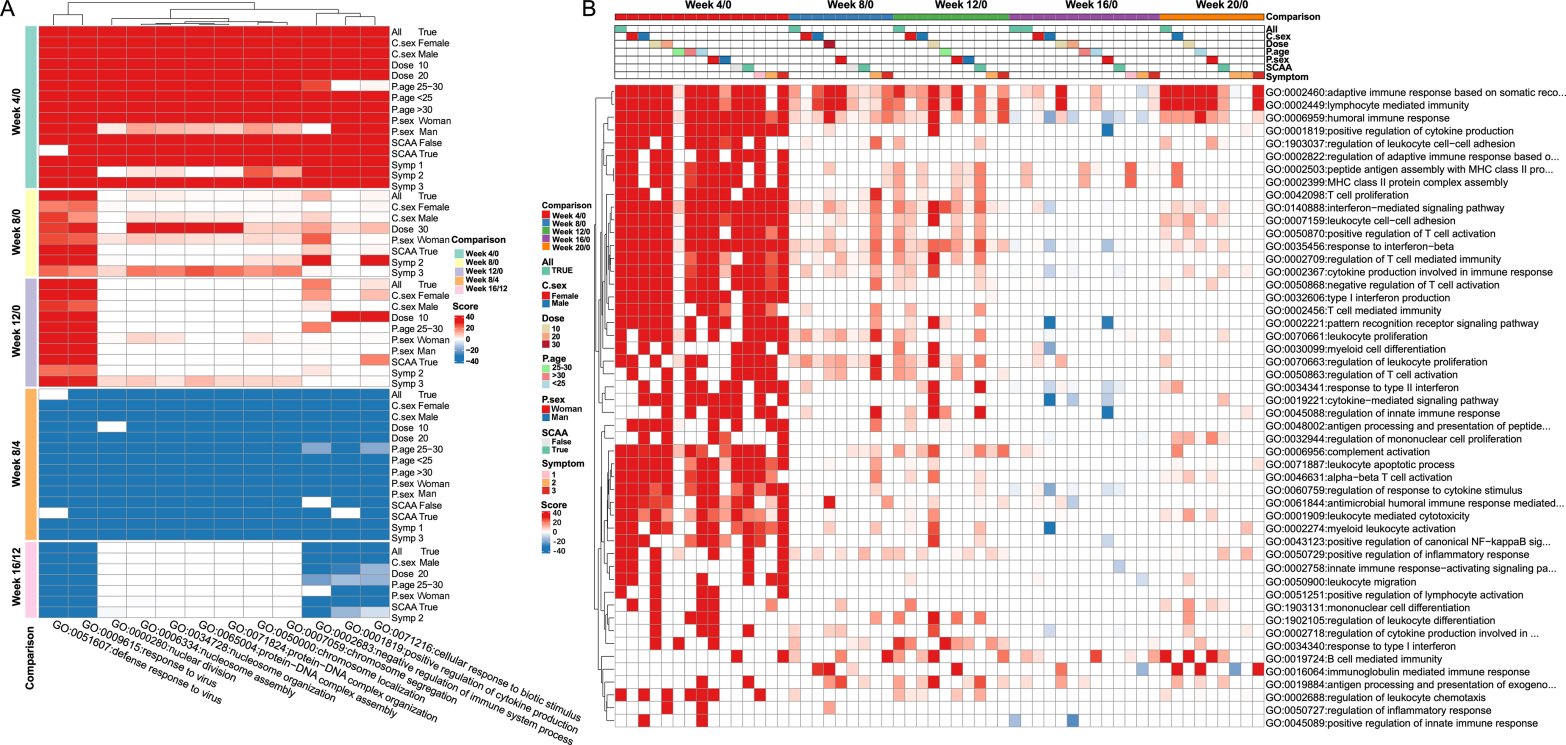
Gene ontology functional analysis of DEGs by gene set enrichment analysis. **(A)** Enriched GO terms repeatedly detected across time points and subgroups. Upregulated and downregulated terms are shown in red and blue, respectively. **(B)** Temporal dynamics of immunity-related GO terms. Color score reflects -log_10_(*P_adj_*)×10; red and blue indicate positive and negative enrichment, respectively.

GO terms that were recurrently enriched across different subgroups were compiled to identify key responses following *S. mansoni* challenge (see **Supplementary Methods**). **Figure 3A** listed the top 12 GO terms repeatedly identified across different subgroups at Week 4 compared to Week 0. Two major categories of upregulated GO terms were enriched in Week 4/0, including several immune (*e.g.*, GO: 0051607, GO:0009615) and cell cycle (e.g., GO:0000280, GO: 0006334) related GO terms, highlighting their importance during the earlier responses to *S. mansoni* challenge. Compared to Week 4, almost all GO terms were downregulated at Week 8 (Week 8/4) and returned to baseline levels (Week 8/0). Even though, two virus-related GO terms remained upregulated at Week 8 when compared to Week 0 (Week 8/0). No recurrently enriched GO terms were identified from downregulated DEGs in either Week 4/0 or Week 8/0, indicating distinct temporal dynamics in pathway regulation. These results suggest that activity in the associated pathways peaked at Week 4 and declined thereafter, consistent with the progression of the *S. mansoni* lifecycle in the human host.

### 3.6 Temporal changes of immune-related pathways after *S. mansoni* cercariae challenge

Although the immune mechanisms underlying *S. mansoni* infection remain incompletely understood, previous studies using animal models suggest that a T-helper 1 (Th1)-dominant immune response occurs during the early migration phase (Weeks 1–5 post-infection). Around Weeks 5-6, as the worms mature, pair, and begin laying eggs, the immune response is thought to shift toward a Th2-dominant profile (25). As expected, our results also revealed that changes in DEGs and immune-related pathways were strongly time-dependent, mirroring the lifecycle of *S. mansoni* in the human host.

To examine transcriptional changes specifically associated with Th1- and Th2-related pathways, we tracked the temporal changes in GO terms related to T-helper cell responses. Compare to baseline, several GO terms related to T cell proliferation (GO:0042098, NES = 2.27, *P_adj_* = 4 × 10^-9^), regulation of T cell activation (GO:0050870, GO:0050868, GO:00466331), regulation of T cell mediated immunity (GO:0002456, GO:0002709), and type 2 immune response (GO:0042092, *P_adj_* = 0.007) were significantly upregulated at Week 4. No GO term directly related to Th1 response was identified, while only one GO term related to Th2 immune response was determined. This suggests that Th2 responses might occur at earlier stage of the infection even though no egg was produced. Moreover, these upregulated GO terms returned to baseline levels in the following weeks except in the subgroup Symp:2, in which most GO terms remained upregulated at Week 8.

To gain deeper insight into the temporal dynamics of immune activity, we broadened our GO term search using a wider range of keywords reflecting diverse immune processes. This approach revealed a greater number of immune-related GO terms, with the top 50 summarized in **Figure 3B**. Overall, most GO terms were significantly upregulated at Week 4 relative to baseline and progressively declined to baseline levels over subsequent weeks. These terms covered both innate and adaptive immune processes. Among them, the top three GO terms were adaptive immune response based on somatic recombination of immune receptors built from immunoglobulin superfamily domains (GO:0002460), lymphocyte-mediated immunity (GO:0002449), and humoral immune response (GO:0006959). These findings emphasize the early engagement of adaptive immunity in response to *S. mansoni* exposure. Additional GO terms reflected leukocyte proliferation (GO:0070661, GO:0070663), cell-cell adhesion (GO:1903037, GO:0007159), activation (GO:002274), and apoptosis (GO:0071887). Multiple GO terms related to T cell proliferation, activation, and regulation were also observed (e.g., GO:0042098, GO:0050870, GO:0002709), underscoring the central role of T cell-mediated immunity.

We also identified GO terms related to MHC class II-mediated antigen processing and presentation (GO:0002503, GO:0002399), along with numerous cytokine- and chemokine-associated terms crucial for immune cell signaling, differentiation, and trafficking. This included regulation of cytokine production (GO:0001819, GO:0002367), response to type I and II interferons (GO:0035456, GO:0034341), regulation of cytokine-mediated signaling pathway (GO:0019221), and response to cytokine stimulus (GO:0060759). Other upregulated GO terms at Week 4 included complement activation (GO:0006956), positive regulation of canonical NF-kappaB signaling (GO:0043123), positive regulation of inflammatory response (GO:0050729), and pattern recognition receptor signaling pathway (GO:0002221). In summary, exposure to *S. mansoni* cercariae rapidly triggered a robust and diverse immune response, as reflected by transcriptional changes at Week 4. These immune responses began to wane in the following weeks, returning closer to baseline.

### 3.7 Construction of protein-protein interaction networks and identification of hub genes

To better understand the functional interconnectedness of the identified DEGs, protein-protein interaction (PPI) networks were constructed using the STRING database. Given that both DEG and GO enrichment analyses indicated the most significant transcriptomic changes occurred at Week 4, we first constructed the PPI network using upregulated DEGs from that time point (**Supplementary Figure S15**). The network revealed three major functional modules, including (a) genes involved in cell cycle regulation; (b) histone-related genes associated with DNA packaging, gene expression regulation, DNA repair, and cell cycle control; and (c) genes involved in various immune responses.

**Figure 4:**
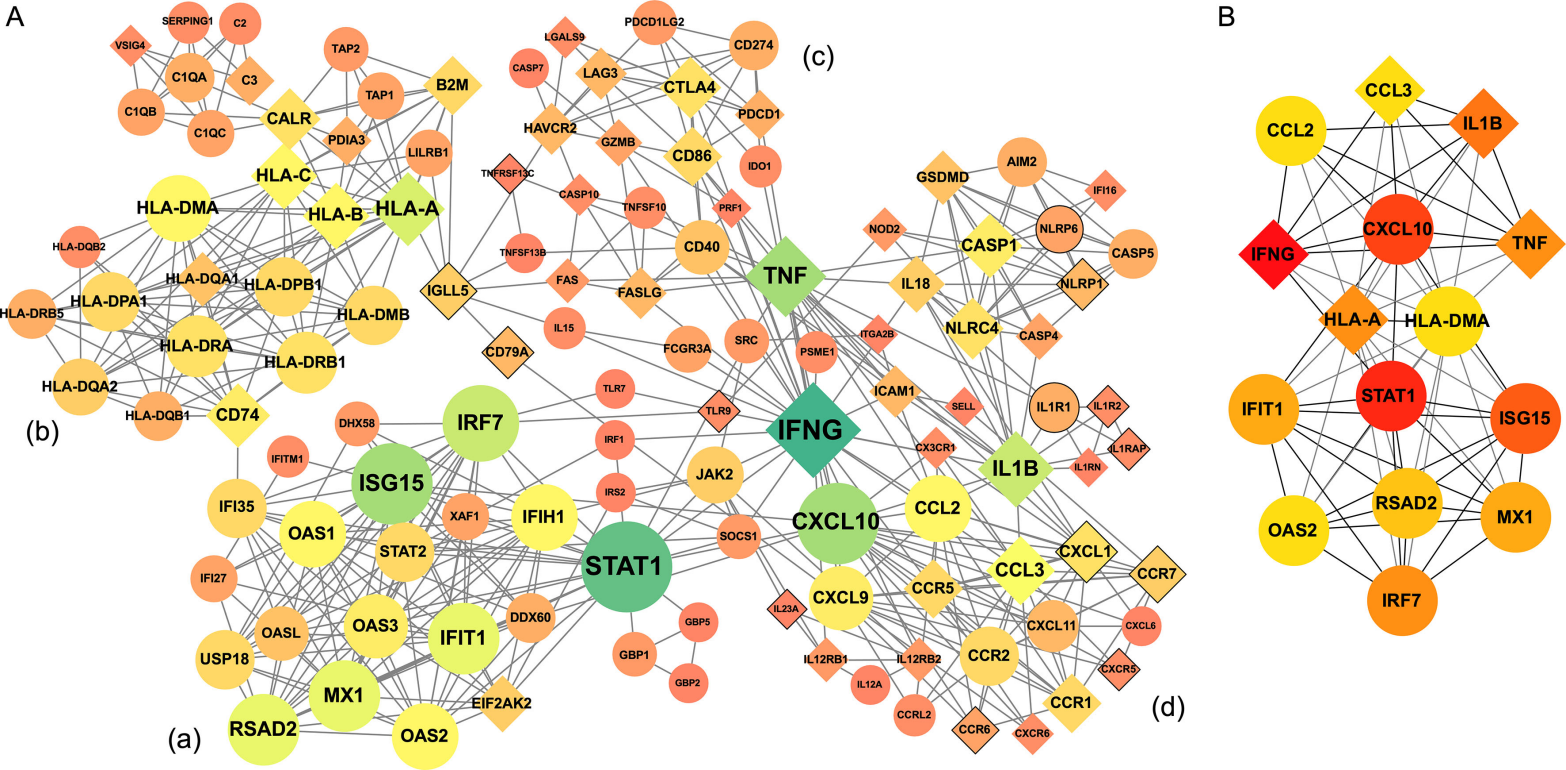
Protein-protein interaction network construction and identification of hub genes. **(A)** Protein-protein interaction network of immune-related DEGs constructed using the STRING with four key modules identified (a-d). Larger and darker nodes indicate higher connectivity. **(B)** Top 15 hub genes ranked by degree. Circles represent genes differentially expressed in all samples. Diamonds denote genes specific to subgroups; black solid outlines represent genes that are down regulated.

We then focused on a subnetwork composed of DEGs related to immune responses at Week 4. This targeted PPI network was constructed using 123 DEGs, the majority of which were upregulated and contributed to the GO terms enriched in **Figure 3B**. Some of these genes were DEGs only in specific subgroups, but not in all samples at Week 4. For example, *IFNG* was significantly upregulated in subgroups P. age: <25 and C. sex: female, while *HLA-A*, *HLA-B*, *IL1B*, *TNF*, and *CD86* were upregulated in subgroup Symp: 3. The immune-focused network included 123 nodes and 485 edges. Four key modules were identified (**Figure 4A**): Module (a) relates to innate antiviral immunity and includes many interferon-stimulated genes (e.g., *ISG15*, *IFIH1*, *OAS2*, *MX1*) and regulators of interferon production (*STAT1*, *STAT2*, *USP18*, *IRF7*). Module (b) contains proteins from both HLA (MHC) class I and II families (e.g., *HLA-DMA*, *HLA-DPB1* from class II; *HLA-A*, *HLA-B* from class I). Genes involved in MHC class II pathways were strongly upregulated in all participants, while MHC class I genes were mainly upregulated in subgroup Symp:3, suggesting class I antigen presentation might be specific to or enhanced in this subgroup. Module (c) includes genes linked to apoptosis (e.g., *TNFSF10*, *CASP7*) and T cell activation and regulation (e.g., *IDO1*, *CD274*). Many DEGs in this module, *GZMB*, *CD86*, *CASP10*, *FASLG*, *HAVCR2*, *LGALS9*, *PRF1*, were mainly upregulated in Symp:3, indicating possible activation of apoptosis and immune suppression pathways in this subgroup. Module (d) is enriched for cytokines and chemokines involved in immune cell recruitment, migration, and activation. For instance, CXCL9, CXCL10, and CXCL11 are CXC chemokines induced by IFN-γ that regulate immune cell migration and differentiation (naïve T cells to Th1 cells) (26). The CC chemokine CCL2 and its receptor CCR2 form the CCR2/CCL2 axis, which promotes cell migration and regulates cell adhesion and macrophage chemotaxis (27). Similar to Module (c), many genes in Module (d) were mainly differentially expressed in Symp:3. Among these, some were upregulated (*IL1B*, *CXCR6*, *CX3CR1*, *CCR1*, *CCR5*, *CCR7*, *CCL3*, *SELL*), while others were downregulated (*CCR6*, *CXCR5*, *CXCL1*, *TLR9*).

In addition to the four main modules, smaller clusters were also observed. Members of the GBP family (*GBP1*, *GBP2*, *GBP5*), encoding interferon-induced GTPases, were connected to other modules via *STAT1*. Complement system proteins (e.g., C1q, C2) were linked to Module (b), indicating potential roles in host defense against *S. mansoni*. *C3* was mainly upregulated in several subgroups, including Symp:3, SCAA: true, and P.sex: woman. Genes related to inflammasome formation and inflammatory responses were also observed near Module (d), linked through *IL1B*, with many of these DEGs derived from Symp:3, suggesting stronger proinflammatory responses in this subgroup.

The PPI network highlights how different modules are connected through key hub genes. Fifteen genes (**Figure 4B**) were identified as hub genes using the CytoHubba tool, including *IFNG*, *STAT1*, *CXCL10*, *ISG15*, *IL1B*, *TNF*, *IRF7*, *HLA-A*, *IFIT1*, *MX1*, *RSAD2*, *CCL2*, *CCL3*, *HLA-DMA*, and *OAS2*. Notably, *IFNG*, *IL1B*, *TNF*, *HLA-A*, and *CCL3* were not DEGs at all participants level but were upregulated in subgroup such as Symp:3, highlighting possible subgroup-specific immune dynamics at Week 4.

### 3.8 Identification of synchronous gene regulation by fuzzy clustering

**Figure 5:**
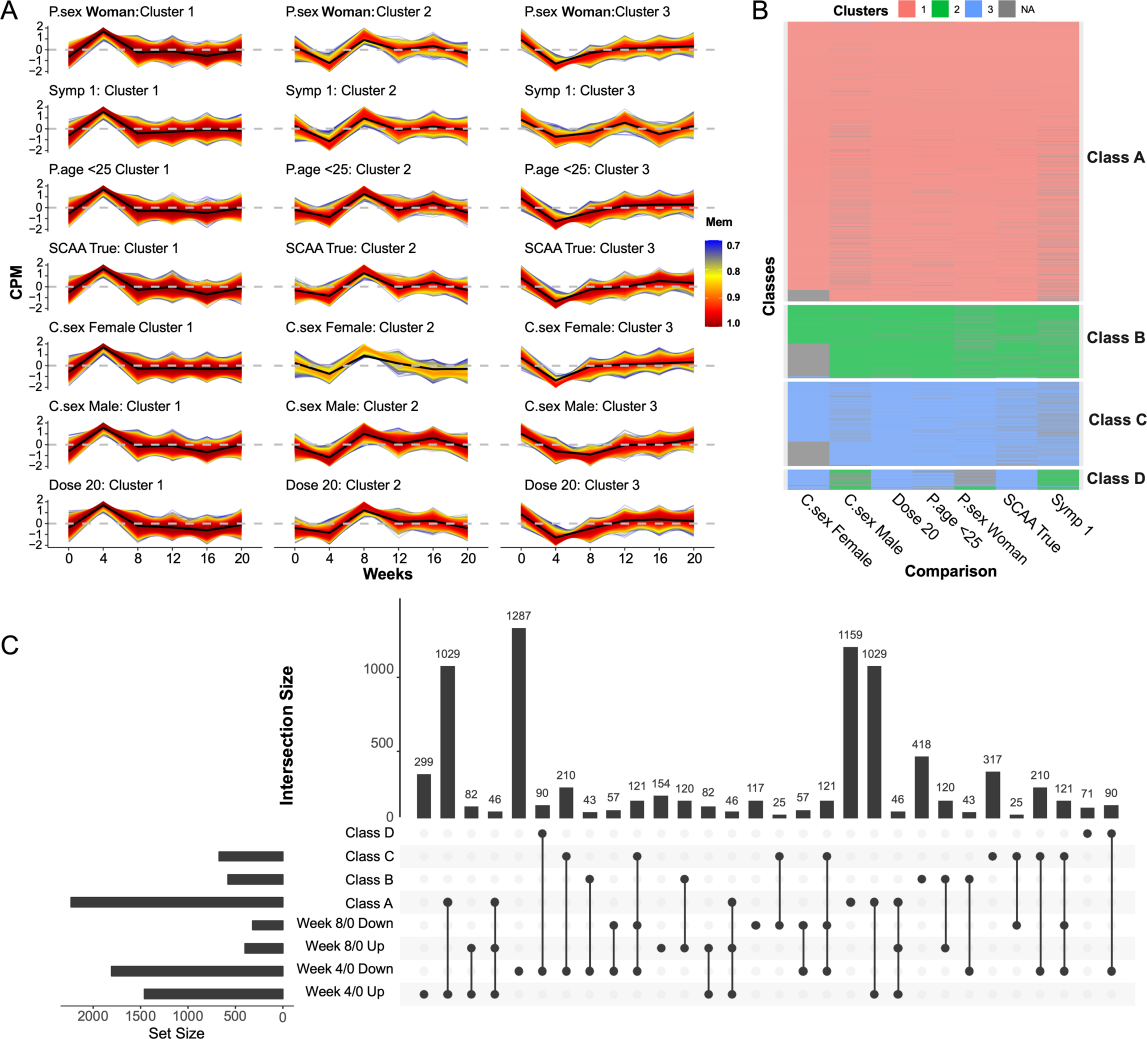
Fuzzy clustering of gene expression in subgroups with >10 women participants. **(A)** Fuzzy clustering of genes in each sample subgroup. Three distinct gene clusters were identified across six time points. Each thin line represents an individual gene, with color indicating membership score (only genes with scores > 0.7 are shown). Red lines highlight genes with high membership values. The dark line shows the average expression profile for each cluster; cluster centers are indicated in black. Rows correspond to different subgroups. **(B)** Meta-clustering of genes. Four gene classes were identified via a second round of clustering, based on cluster labels from fuzzy clustering across all subgroups. **(C)** UpSet plot showing intersections between meta-cluster gene classes and DEGs at Weeks 4 and 8. The dot matrix indicates the presence of gene sets in specific intersections, with vertical lines connecting intersecting sets. The left bar chart displays the size of each gene set, and the top bar chart shows the size of each intersection.

Genes involved in the same biological pathway are often co-regulated by shared transcription factors, leading to coordinated expression changes over time (28). To identify genes exhibiting synchronous expression changes following *S. mansoni* cercariae challenge, fuzzy clustering was applied to subgroups with at least 10-woman participants. Based on the elbow method for determining the optimal number of clusters (see **Supplementary Methods**), three clusters were identified for each subgroup (**Figure 5A**, **Supplementary Table 5**). Cluster 1 genes showed strong activation, peaking at Week 4, then returned to baseline at Week 8, which stabilized thereafter. In contrast, Cluster 2 genes were suppressed at Week 4, then upregulated with a peak at Week 8, followed by a gradual decline. Cluster 3 genes also showed their lowest expression at Week 4, after which transcriptional levels steadily increased through Week 20.

The adjusted Rand index (ARI) indicated that gene clustering patterns across subgroups were highly consistent (ARI > 0.95, **Supplementary Figure S16**). To identify genes consistently clustered together across subgroups, a second round of clustering (meta-clustering) was performed based on the cluster labels from fuzzy clustering, resulting in four distinct gene classes (**Figure 5B**, **Supplementary Table 5**). Gene Class A, B, and C primarily corresponded to Clusters 1, 2, and 3 in each subgroup, respectively, while Class D comprised genes that were inconsistently clustered across subgroups. Intersection of gene categories with DEGs showed that 1,159 of 2,234 genes were unique to Class A, 418 of 581 to Class B, 317 of 673 to Class C, and 71 of 161 to Class D (**Figure 5C**). These findings suggest that fuzzy clustering can identify additional synergistically expressed genes not captured by DEG analysis.

**Figure 6:**
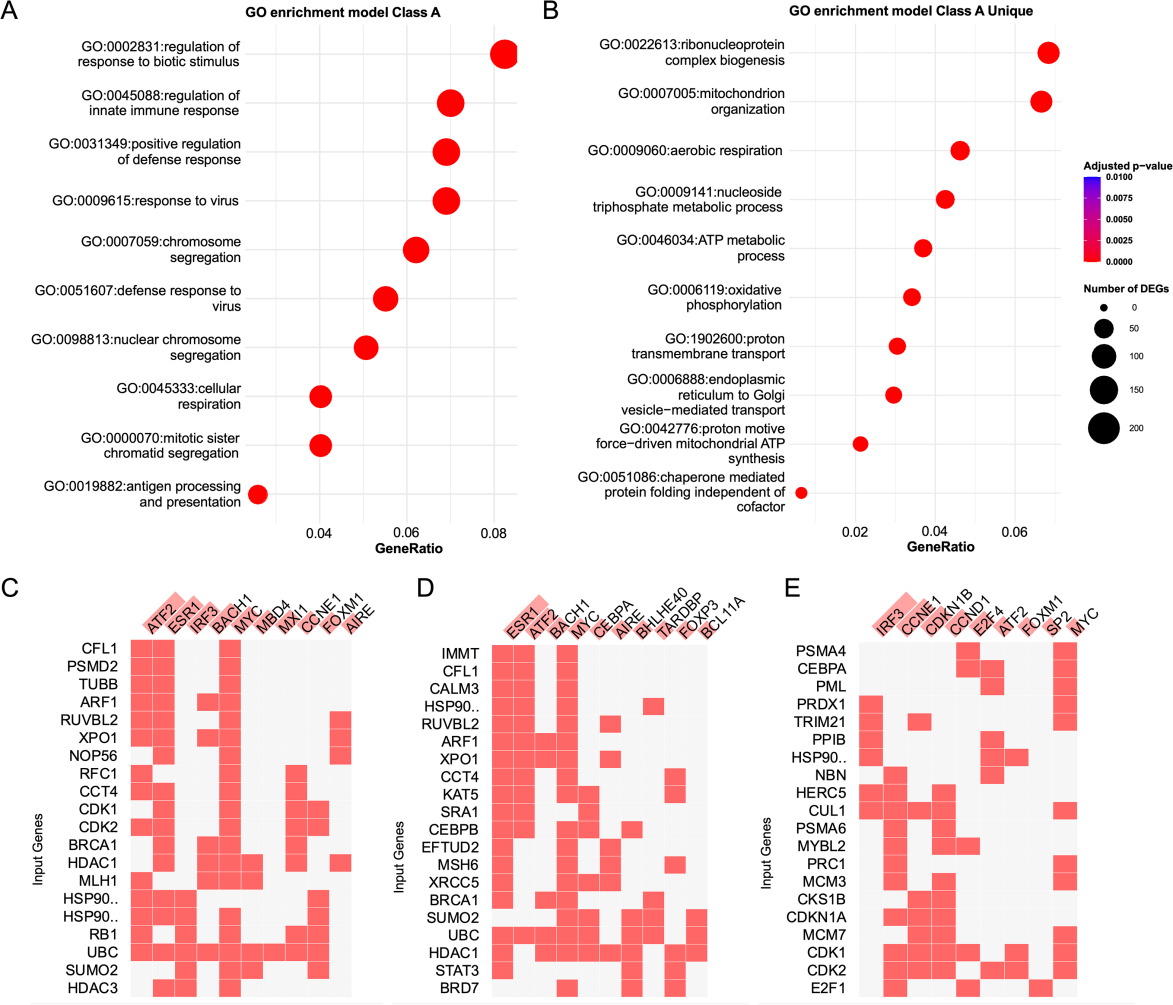
Gene ontology enrichment for Class 1 genes and identification of transcription factors. **(A, B)** Top 10 enriched GO terms for Class 1 genes **(A)** and Class 1-unique genes **(B)**. **(C–E)** Dot size indicates the number of genes per term; color reflects adjusted P value significance. Heatmaps of predicted transcription factors regulating: **(C)** Class 1 genes, **(D)** Class 1-unique genes, and **(E)** genes shared between Class 1 and DEGs. Only the top 10 transcription factors are shown. Red cells indicate predicted regulation of the corresponding gene by each transcription factor. Transcription factors are ranked by combined score, represented by background bar length behind each name.

GO enrichment analysis revealed that only Class A genes were significantly enriched for GO terms (**Figure 6A**, **Supplementary Table 6A**). The most upregulated GO term was GO:0009615 (response to virus, *P_adj_* = 8 × 10^-26^). The top ten enriched terms indicated that Class A genes are primarily involved in immunity (e.g., GO:0002831, GO:0045088), energy metabolism (GO:0045333), and cell division (e.g., GO:0007059, GO:0098813). Enrichment analysis of Class A-unique genes (those not identified as DEGs) highlighted biomacromolecule synthesis and energy metabolism (e.g., GO:0007005, GO:0022613) as dominant biological processes (**Figure 6B**, **Supplementary Table 6B**), and the most upregulated GO term was GO:0009060 (aerobic respiration, *P_adj_* = 2 × 10^-13^). These findings suggest that immunity- and cell cycle-related genes are synchronously regulated and exhibit significant changes at Week 4 post cercarial exposure. While some of these genes were also detected by DEG analysis, fuzzy clustering further revealed additional genes related to material and energy metabolism that likely support the immune response. Similarly, transcription factor target enrichment analysis yielded notable results only for Class A. The top ten enriched transcription factors included *ATF2*, *ERS1*, *IRF3*, *BACH1*, *MYC*, *MBD4*, *MXI1*, *CCNE1*, *FOXM1*, and *AIRE*. Specifically, *ESR1* and *ATF2* were most significantly enriched among Class A-unique genes, whereas *IRF3* and *CCNE1* were primarily enriched among genes shared between DEGs and Class A (**Figures 6C-E**, **Supplementary Table 7**).

### 3.9 Construction of co-expressed gene modules associated with experimental factors

**Figure 7:**
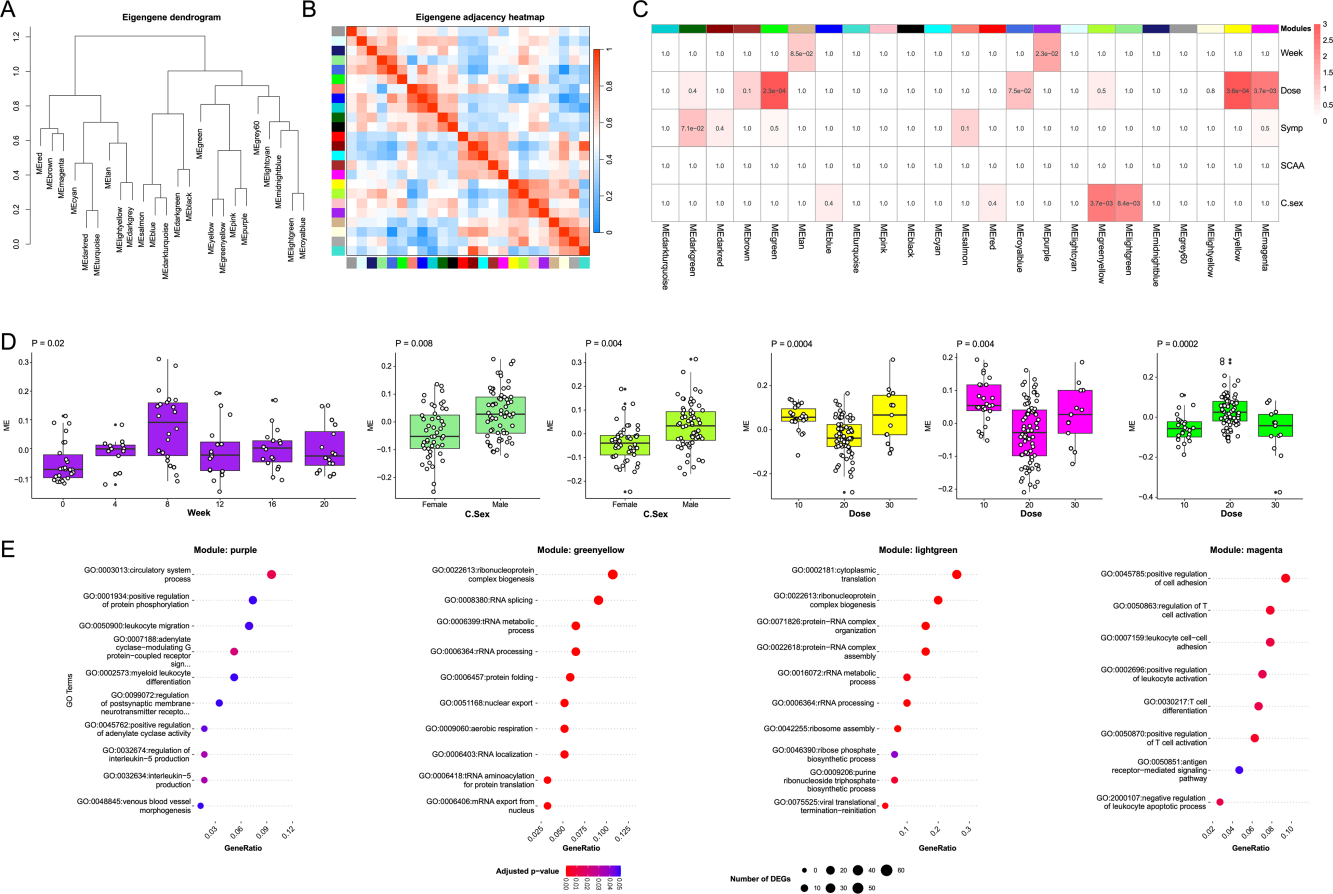
Weighted Gene Co-expression Network Analysis. **(A)** Dendrogram showing clustering of 23 identified gene co-expression modules. **(B)** Heatmap of eigengene adjacency showing relationships between module eigengenes and experimental traits. **(C)** ANOVA of module eigengene expression across participant groups stratified by experimental variables. Cell color indicates -log_10_(*P*_adj_). **(D)** Boxplot of eigengene expression in different color modules including purple, green yellow, light green, and magenta. **(E)** GO enrichment analysis of modules associated with time (weeks), dose, and cercariae sex.

WGCNA was employed to detect modules of highly correlated genes potentially associated with experimental traits. As shown in **Figures 4A, B**, a total of 23 modules were identified, with module sizes ranging from 33 to 1,776 genes (**Supplementary Figure S17A**, **Supplementary Table 8**). Analysis of variance (ANOVA) of module eigengene expression revealed that the purple module exhibited significant variation across time points, with peak expression observed at Week 8 (**Figures 7C, D**). Intersection analysis showed that 17% of genes in the purple module overlapped with upregulated DEGs at Week 4, while 66% overlapped with upregulated DEGs at Week 8 (**Supplementary Figures S17B-E**). GO enrichment analysis indicated that genes in the purple module were predominantly involved in leukocyte migration through the circulatory system, and the most enriched GO term was GO:0003013 (circulatory system process, *P_adj_* = 0.01) (**Figure 7E**, **Supplementary Table 9**). Furthermore, the expression levels of two modules, light green and green yellow, were associated with the sex of cercariae (**Figures 7C, D**). GO enrichment analysis suggested that both modules were involved in rRNA processing and protein synthesis (**Figure 7E**, **Supplementary Table 9**), and the most enriched GO term was GO:0002181 (cytoplasmic translation, *P_adj_* = 1 × 10^-25^) in light green and GO:0022613 (ribonucleoprotein complex biogenesis, *P_adj_* = 8 × 10^-6^) in yellow green. Despite their functional similarity, eigengene adjacency analysis showed that these modules belong to distinct module groups (**Figure 7B**). These findings imply that the intrinsic sexual differences of cercariae may induce distinct cellular anabolic responses. Additionally, three modules, yellow, magenta, and green, showed expression differences among groups exposed to varying cercarial doses (**Figures 7C, D**). Of these, only magenta modules exhibited significantly enriched GO terms (**Figure 7E**, **Supplementary Table 9**). The magenta module was primarily associated with T cell regulation (the most enriched GO term was GO:0045785, positive regulation of cell adhesion, *P_adj_* = 0.02).

### 3.10 Blood transcriptome module enrichment over time

**Figure 8:**
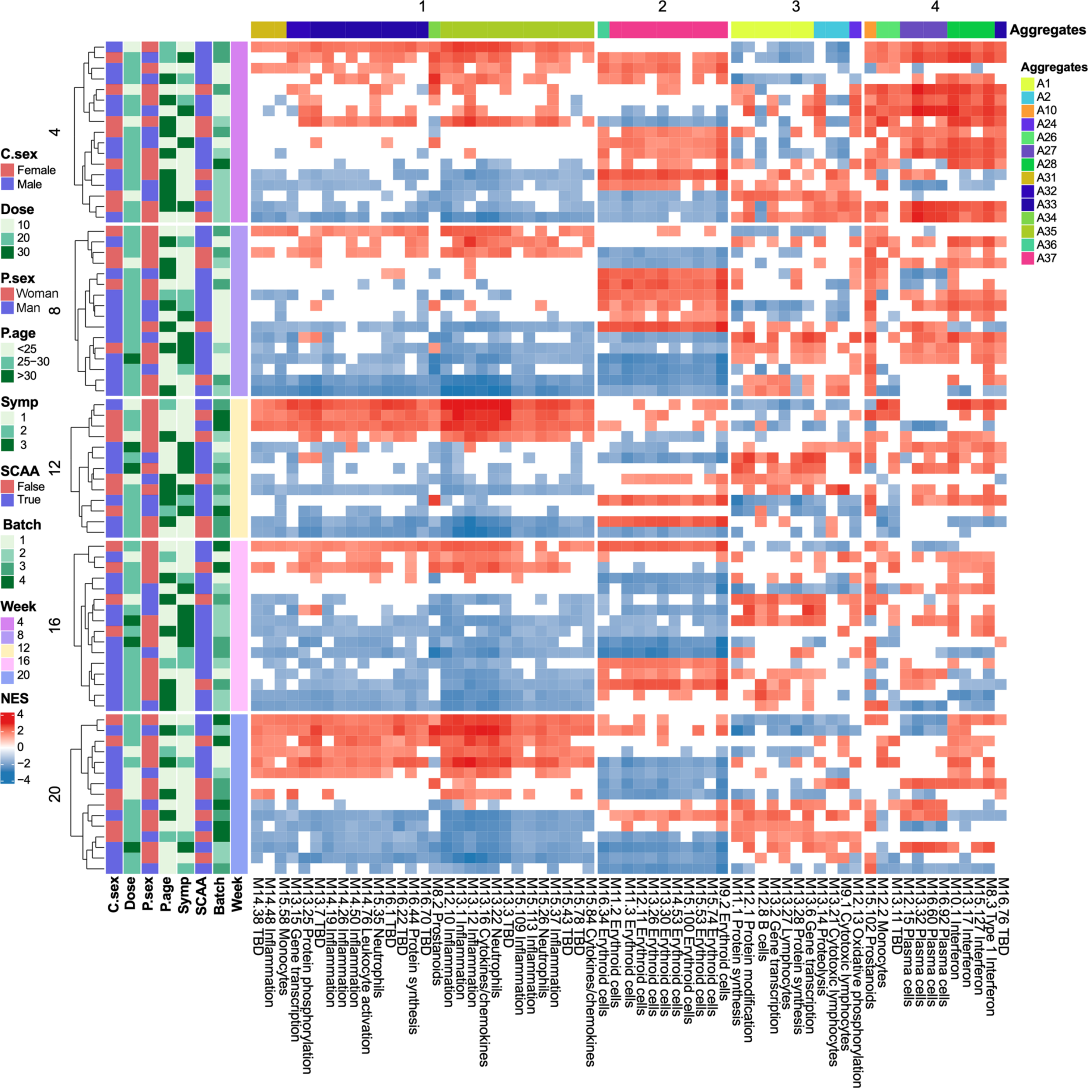
BloodGen3Module gene expression dynamics in individual participants. Only samples with normalized enrichment score *P* < 0.05 are shown. Columns represent participants grouped by cluster assignment; rows represent genes grouped by functional modules. Heatmap shows individual-level fingerprints of transcript abundance for individual modules (columns) belong to different aggregates across individual participants (rows). Rows are arranged by time point. Red indicates increased transcript abundance compared to baseline (Week 0), with proportions ranging from +15% to +100%. Blue indicates decreased abundance, ranging from -15% to -100%.

In addition to group-level transcriptional changes, we also assessed transcriptional variation within individual participants using BloodGen3Module. Each BloodGen3 module consists of a set of genes ranging from 12 to 169, with an average of 37 genes per module. **Figure 8** shows heatmaps of normalized enrichment scores for significantly enriched modules, where red and blue indicate up- and downregulation, respectively. The heatmap revealed substantial inter-individual variability, suggesting non-uniform immune responses to cercariae exposure among participants. We clustered 63 enriched modules into four clusters based on similar longitudinal expression patterns: Cluster 1 included 30 modules from aggregates A26, A31, A33, A34, and A35, with A33 and A35 particularly associated with inflammation. In some individuals, these modules remained upregulated from Week 4 to Week 20; in others, they were suppressed. Cluster 2 included two aggregates, A36 and A37, with all 11 modules related to erythroid cells. This cluster showed a shift from positive enrichment at Week 4 to predominantly negative enrichment by Week 20. Cluster 3 included 11 modules from aggregates A1, A2, and A24, representing lymphocytic responses and oxidative phosphorylation. No consistent temporal pattern was observed across participants. Cluster 4 composed of 11 modules from aggregates A10, A26, A27, A28, and A33. Specifically, A27 was associated with “cell cycle” with four modules related to plasma cells, which are linked to the presence of antibody-producing cells in peripheral blood. A28 was associated with “interferon”, with four modules detected, including one related to type I interferon. Not surprisingly, modules in Cluster 4 were positively enriched across all individuals at Week 4 and remained upregulated, though at lower intensity, at Week 8, followed by a gradual decline in enrichment thereafter.

## 4 Discussion

In the current study, we investigated temporal changes in gene expression occurring in the host immune system due to single sex male- or female-only cercarial challenge. Our understanding of early host responses and underlying mechanisms against *S. mansoni* infectious larvae remain limited. Therefore, in this report, we comprehensively analyzed RNA-Seq data from two single-sex CHI trials, for the first time profiling transcriptional changes in 30 participants over 20 weeks following *S. mansoni* cercarial challenge. Our analyses have identified key genes and immune-related pathways that may be actively involved in the early phase of infection.

Time-dependent variation in immune-related transcriptional responses following the infectious challenge appeared to be the main component. Both PCA and DEG analyses showed that Weeks 4 and 8 deviated from other time points, suggesting that immune responses to *S. mansoni* cercarial challenge at these two stages may be distinct. These results also indicate that host immune responses closely align with different stages of the *S. mansoni* life cycle. Transcriptional changes observed at Week 8 appear to correspond to immune reactions against fully developed worms, whereas changes at Week 4 likely reflect the host’s response to immature larval worms and/or worms transitioning to adulthood. These findings are consistent with observations from our CHI trials. Therefore, further transcriptomic profiling at these two time points may provide crucial insights into the immune mechanisms underlying acute schistosomiasis caused by *S. mansoni*.

Compared to the transcriptional profile at pre-challenge (Week 0), Week 4 had the highest number of DEGs and enriched GO terms, suggesting that the immune response around this time may be the most robust among all time points examined. Although many downregulated DEGs were identified, very few were associated with enriched GO terms. These downregulated genes likely reflect regulatory changes that support immune activation (24). On the contrary, upregulated DEGs at Week 4 were associated with numerous immunity-related GO terms, highlighting the complexity of the host immune responses against *S. mansoni* cercarial challenge. Together with PPI network results, our data indicated that distinct immune responses were actively engaged at this stage. A mixed Th1/Th2 immune response even before the onset of egg-laying is known to occur (29–31). Similar to CHI trial observations, the identification of Th2-related GO term at Week 4 reflected the activation of Th2 responses at earlier stages of the infection, which supports the view that Th2 responses can occur at earlier stages of infection, even in the absence of egg deposition. In addition to DEGs associated with a wide range of immune responses, *S. mansoni* cercariae challenge also resulted in strong alteration of many genes related to other processes such as cell cycle progression and proliferation at Week 4, as further revealed by enriched GO terms. There have been some previous *in vitro* and *in vivo* studies showing that infection by *Schistosoma* could influence cell proliferation and cell cycle status (22, 23, 32–35). However, the differences in study designs (e.g., parasite species, infection status, and type of cell lines, organ, and animal model) and research goals as well as the potential complex underlying mechanisms make the comparison among these available studies challenging.

Several critical genes that may play important functional roles during the initial migration stage post-challenge were identified via DEG analysis and PPI network construction. Among the top upregulated DEGs at Week 4, many are known to have essential roles in the immune system. The most highly upregulated gene was *LINC02528* or *SIMALR*. This non-conserved, human macrophage-specific lincRNA is highly induced in lipopolysaccharide/interferon-γ (LPS/IFN-γ)-stimulated macrophages and may help regulate macrophage survival by protecting against inflammation induced apoptosis (36). *CXCL10* and *CXCL9* were the second and fourth most upregulated genes, respectively, and *CXCL11* was also significantly upregulated. These three genes encode Th1-associated chemokines that are strongly induced by IFN-γ (37, 38). All three bind to the same primary receptor, CXCR3, and are involved in promoting T cell adhesion, migration, and activation, as well as in the chemoattraction of monocytes, macrophages, T cells, natural killer (NK) cells, and dendritic cells (37, 39, 40). Increased IP-10 (CXCL10) levels were detected in both CHI trials, peaking at Week 4 and declining thereafter, consistent with our DEG findings (16, 17). Individuals from schistosomiasis-endemic regions exhibit higher levels of CXCL10 and CXCL9 compared to non-infected individuals (41). Furthermore, *CXCL10* was identified as a hub gene in our PPI network, further emphasizing its central role in the early immune response to *S. mansoni* infection.

The third most upregulated gene, *APOL4*, belongs to the human apolipoprotein L (*APOL*) gene family, which comprises six isoforms (42). Four other *APOL* genes, including *APOL1*, *APOL2*, *APOL3*, and *APOL6*, were also significantly upregulated. The APOL genes are interferon regulated and are typically induced by proinflammatory signals. Our current understanding of the precise biochemical functions of proteins in the APOL family remains limited.

Genes belonging to the guanylate-binding protein (GBP) family, including *GBP1*, *GBP2*, *GBP3*, *GBP4*, and *GBP5*, were also strongly upregulated at Week 4. GBPs are a family of dynamin-related large GTPases expressed in response to interferons and other proinflammatory cytokines. They participate in a wide range of innate immune functions against intracellular pathogens (43). A recent study reported that *GBP4* was significantly upregulated in bone marrow-derived macrophages from *S. mansoni*-infected male mice (44). Another significantly upregulated gene family was the human leukocyte antigen (HLA) class II, which are essential for initiating T cell mediated adaptive immune responses (45, 46). Additionally, PPI analysis identified *HLA-DMA* as a key hub gene, and two GO terms related to MHC class II assembly and function (GO:0002503 and GO:0002399) were significantly enriched at Week 4.

Among other upregulated DEGs, *ATF3*, *CFB*, and *ACOD1* play vital roles in regulating immune responses. *ATF3* encodes activating transcription factor 3, *ATF3* has also been associated with fibrosis. *CFB* encodes complement factor B, a component of the alternative complement pathway. It is paralogous to *C2*, and both are key regulators of the innate immune system (47). Both GO enrichment analysis (e.g., GO:0006956: complement activation) and PPI network results (including clusters of complement-related genes such as *SERPING1*, *C1QA-C*, *C2*, and *C3*) indicated activation of the complement system at Week 4. These findings suggest that complement pathway activation may be triggered following *S. mansoni* cercarial challenge. Parasites, including schistosome, are known to evade or subvert complement mediated attack (48, 49). Notably, *C3* was only upregulated in certain subgroups, including Symp:3, suggesting potential subgroup specific complement activation, although further studies are needed to elucidate if complement play definitive role in creating membrane attack formation on the pathogen.

We have also identified 15 important genes acting as “hubs” in the PPI network, including *IFNG*, *STAT1*, *CXCL10*, *ISG15*, *IL1B*, *TNF*, *IRF7*, *HLA-A*, *IFIT1*, *MX1*, *RSAD2*, *CCL2*, *CCL3*, *HLA-DMA*, and *OAS2*. These hub genes are known to play essential roles across a wide range of immune responses. *IFNG*, which encodes interferon-gamma (IFN-γ), the only type II interferon, ranked as the top hub gene by degree. IFN-γ is a well-established key regulator of host defense, mediating both innate and adaptive immune responses. Both GO enrichment and PPI analyses support a central role for IFN-γ in the early stages following *S. mansoni* exposure. Furthermore, the GO term GO:0034341 (response to type II interferon) was upregulated in most subgroups at Week 4. The PPI network also shows that IFN-γ directly or indirectly influences many other critical immune molecules, such as CXCL9-11. Additionally, several genes within the IFN-γ signaling pathway, including *STAT1* and *JAK2*, were upregulated at Week 4, further emphasizing the importance of this pathway. Interestingly, *IFNG* itself was not differentially expressed in all participants at Week 4. Instead, it appeared as a DEG only in two subgroups, including P.age < 25 and C.sex: female. We therefore propose that the upregulation of *IFNG* may have occurred at an earlier time point and sampling at Week 4 may have missed this transient expression peak. Many previous studies have examined changes in IFN-γ during schistosomiasis, with some reporting that acute schistosomiasis is predominantly associated with Th1 responses and increased levels of IFN-γ, which decline as Th2 responses become predominant (50–52). Consistent with these findings, the sustained upregulation of genes regulated by or associated with IFN-γ observed in our study suggests that IFN-γ plays a critical role in orchestrating immune responses to *S. mansoni* infection.

Many interferon-related genes such as *STAT1*, *IFIT1*, *ISG15*, *IRF7*, *MX1*, *RSAD2*, *OAS2*, as well as *CXCL10* discussed earlier, were also identified as hub genes in the PPI network, further highlighting the importance of interferon-related responses during the early phase of *S. mansoni* infection. *STAT1* was also the 14th most upregulated DEG at Week 4. It belongs to the signal transducer and activator of transcription (*STAT*) family and is a critical component of the immune response against pathogens. *STAT1* is the principal mediator of type I, II, and III interferon signaling, as well as IL-27 signaling, and regulates the transcription of hundreds of downstream genes (53, 54). Several interferon-stimulated genes, including *ISG15* (interferon-stimulated gene 15), *IFIT1* (interferon-induced protein with tetratricopeptide repeats), *MX1* (interferon-induced GTP-binding protein), *RSAD2* (also known as *Viperin*), and *OAS2* (2’-5’-oligoadenylate synthetase 2), were also identified as hub genes. Moreover, the transcription factor *IRF7* (interferon regulatory factor 7) was also identified as a hub gene. These genes are known to play crucial roles in host defense, particularly against viral infections. For example, *ISG15* is one of the most significantly induced genes in response to viral infection and was the first identified ubiquitin-like (UBL) protein induced by type I interferon (55, 56). It regulates a wide spectrum of immune-related cellular pathways. The identification of interferon related hub genes aligns well with the results from GO enrichment and BloodGen3Module analyses, reinforcing the hypothesis that interferon signaling plays a pivotal role in the early immune response to *S. mansoni* infection.

Another key group of hub genes includes chemokine related genes such as *CCL2*, *CCL3* (differentially expressed in Symp:3), and *CXCL10*. These genes are central nodes connecting with other chemokines and their receptors in the PPI network. Their presence highlights their essential roles in recruiting, regulating, and activating immune cells during the host response to *S. mansoni* infection. Finally, the proinflammatory cytokines *TNF* and *IL1B* were also identified as hub genes but were primarily upregulated in the Symp:3 subgroup. TNF (tumor necrosis factor) is an early and key proinflammatory cytokine produced by activated immune cells in response to a wide range of pathogens. There have been many studies working on the role of TNF-α during *S. mansoni* infection, and some investigated how it might affect worm development, metabolism, egg-laying, parasite gene expression, and protein phosphorylation (57–62). However, many of these findings have been inconsistent, suggesting that the influence of TNF-α may be complex. Therefore, further investigation is warranted.

Among the various subgroups, Symp:3, comprising participants who experienced the most severe symptoms, stood out as the most distinctive. Several hub genes were primarily upregulated in this subgroup at Week 4, including *TNF*, *IL1B*, *CCL3*, and *HLA-A*. In addition, many other genes were differentially expressed specifically in Symp:3 at this time point as shown in the PPI network analysis. During the two CHI trials, correlations between clinical symptoms and various immunological or molecular parameters were examined; however, only weak associations were identified. For example, participants exhibiting symptoms at Week 4 showed a trend toward elevated CCL4 (MIP-1β) levels compared to asymptomatic individuals. Furthermore, those with acute schistosomiasis syndrome had higher levels of IFN-γ, Th2 cytokine-producing CD4+ T cells, and CD25+FOXP3+ regulatory T cells at Weeks 4 and 8, though not all differences reached statistical significance. No clear correlation was observed between SCAA levels and symptom severity, although one participant who experienced the most severe adverse events had SCAA levels at least seven times higher than others (17). These transcriptomic and immunological observations suggest that host responses to *S. mansoni* infection may vary considerably among individuals, potentially due to underlying genetic differences. However, it is important to note that only four participants in the Symp:3 subgroup was sampled at Week 4. While this small sample size may introduce bias, the distinct expression patterns and hub gene involvement still underscore potentially important biological differences related to symptom severity.

PCA and DEG analyses indicated participant sex-related differences. It has been well known that sex hormones affect immune function, resulting in differences in the immune phenotype and disease response between women and men participants (63, 64). Unexpectedly, no DEG was identified in the subgroup of men participants (n = 11). It is possible that our earlier sampling points (before Week 8) might have missed the peak of transcriptional changes in men participants, or this may be due to relatively smaller sample size of men compared to women which may not be sufficient to fully capture the changes. There is a possibility that cercariae numbers used to expose participants might have influenced the DEGs in men participants. For example, no men participant was exposed to the cercaria dose of 30. However, possibility does exist that host sex may contribute to differences. Sex related differences have previously been reported, for example, in peripheral blood transcriptional profiles in people with or without active *S. haematobium* infection in the endemic rural areas of Tanzania, significantly more DEGs among female participants (65) with active *S. haematobium* infection than male participants have been reported (23). In addition, through PCA of gene expression, distinct differences between female and male children affected by *S. mansoni* from Albert Nile region have also been recorded (19). A study conducted in the school aged children found upregulated gene expression related to fibrosis which is the late stage of infection due to egg lodging in the tissues. Sex-related differences in the human antibody isotype responses to *S. mansoni* and *S. japonicum* adult worm and soluble egg antigens using sera from individuals in Kenya, the Philippines, and from Uganda (66) have been reported. Sex-dependent immune responses among adult participants with similar *S. haematobium* infection intensity in a chronically infected Senegalese population have been postulated to be due to the influence of nonimmunological factors such as sexual hormones (65). Therefore, it is evident that there are host sex-related differences against *Schistosoma* infection, however, current knowledge may not be able to fully explain such differences. The differences in transcriptional changes between men and women observed in our study may provide a new aspect towards explicating the sex-related differences against *S. mansoni* infection. Further study with a larger sample size and improved sampling design, including sample quality control and sequencing processing, may gather more essential information to improve our current understanding towards host sex-related differences.

Beyond differential expression analysis, we also employed Fuzzy clustering to investigate genes that may be co-regulated but not captured by conventional DEG methods. This analysis revealed distinct temporal expression patterns across gene clusters. Importantly, we identified key transcription factors likely regulating these gene groups. For example, in Class A, characterized by upregulation primarily at Week 4, the top transcription factors included *ATF2*, *ESR1*, *BACH1*, *MYC*, *MBD3*, *MXI1*, *CCNE1*, *FOXM1*, *IRF3*, and *AIRE*. *ATF2* is involved in regulating multiple cellular functions in response to stress and is activated by both mitogenic signals (via ERK) and stress signals (via JNK and p38) (67). *ESR1* is responsive to a broad range of stimuli, including estrogen and growth factors (68). *AIRE* is critical for the maintenance of central immune tolerance and regulates the expression of thousands of genes in medullary thymic epithelial cells (69, 70). *IRF3*, similar to *IRF7*, is a key transcriptional regulator of type I interferons and plays an essential role in antiviral responses. Other transcription factors such as *BACH1*, *MYC*, *MXI1*, and *FOXM1* contribute to fundamental cellular processes including the cell cycle, proliferation, metabolism, differentiation, and apoptosis (71–74).

By analyzing transcriptional changes among different participant groups, we have identified key genes and pathways that may play essential roles during the early stages of human immune responses to *S. mansoni* infection. In addition to group-level analyses, we assessed transcriptional changes at the individual level using BloodGen3Module. We observed significant heterogeneity among participants following *S. mansoni* cercarial exposure, suggesting that immune responses may vary substantially due to underlying confounding factors. Nonetheless, module patterns from Cluster 4 revealed shared immune responses, particularly the induction of interferon- and plasma cell-associated genes from Week 4 to Week 8, consistent with the group-level findings.

To summarize, this study represents the first comprehensive transcriptomic analysis of human responses following controlled *S. mansoni* cercarial challenge. We found that immune responses were highly time-dependent, with Week 4 showing the greatest number of DEGs and enriched GO terms. These transcriptional changes mirrored the lifecycle progression of *S. mansoni* in the human host. Notably, a mixed Th1/Th2 response was already evident at Week 4, contrary to the prevailing view that Th2 responses emerge only after egg deposition. Despite the complexity and inter-individual variability, we identified a set of critical genes, including highly upregulated DEGs and central hub genes at Week 4. For instance, early interferon responses or the expression of key hub genes (e.g., *CXCL10*, *STAT1*) could serve as potential biomarkers of protection or guide adjuvant selection in vaccine design. These findings highlight potential targets for therapeutic intervention and may inform vaccine development by identifying early-stage immune signatures indicative of effective host responses.

## Data Availability

All study data are included in the article or **Supplementary Materials**. Additional data supporting the findings of this study are available from the corresponding author upon request. The RNA-seq dataset has been submitted to NCBI GEO and is available under GEO accession number GSE313156.
