# Supplementary material for: Temporal modulation of gene expression in a controlled Schistosoma mansoni human infection model
Source: Front Immunol. 2025 Dec 16;16:1707749. doi: 10.3389/fimmu.2025.1707749 (PMC12748245; doi:10.3389/fimmu.2025.1707749)
Supplement: Supplementary file 1 [file Table1.docx]

Supplementary Materials

Supplementary Methods

FASTQ Raw Read Quality Control: Stringent quality control procedures were applied to the raw FASTQ files to obtain clean reads. Briefly, the FASTQ files were first assessed using FastQC(1) for initial quality checks and examined to verify single-/paired-endedness and strandedness(2, 3). Fastp was then used to trim 5' and 3' low-quality nucleotides (Phred score <20), detect and remove adapter sequences, discard low-quality reads, and eliminate reads deemed too short(4) (parameters set as -detect_adapter_for_pe, -q 20, and -l 20, with all other setting at default). After filtering, reads were analyzed to identify rRNA fragments using SortMeRNA(5) and mapped against metagenomic species using Kraken2/Bracken(6) to detect potential contamination from non-human species. Reads mapped to contaminants were isolated and further examined to determine whether they represented true contamination. Verified contaminant reads (e.g., those exhibiting systematic patterns(7)) were then removed from the dataset. Finally, the cleaned FASTQ files underwent a second round of FastQC, and quality reports were aggregated using MultiQC(8). The final reports were reviewed to confirm that the remaining reads met all established quality standards.

**Read Alignment and Gene Expression Quantification:** Clean reads were aligned to the human reference genome (CHM13) using STAR(9) to generate BAM files. Gene-level read quantification was performed using featureCounts(10). To account for differences in sequencing depth across samples, raw read counts were normalized using the trimmed mean of M-value (TMM) method, as implemented in edgeR(11). The resulting normalized count matrix and log₂ counts per million (log₂CPM) values were used for all downstream analyses. A simplified bioinformatics workflow is shown in **Figure S2**, summarizing steps from pre-alignment quality control to differential expression and pathway analysis.

**Principal Component Analysis (PCA):** To detect potential sample outliers, TMM-normalized log₂CPM values from the previous step were used to construct PCA plots using the PCAtools R package. Principal components were calculated using the pca() function with the scale parameter set to FALSE. The first two components were visualized using the biplot() function. To estimate distribution regions of samples sharing similar characteristics, the ellipse parameter was set to TRUE, assuming a multivariate t-distribution.

**Differential Gene Expression Analyses:** Differential gene expression analyses were performed using edgeR(11) to identify differentially expressed genes (DEGs) across various comparisons. To examine the transcriptional response to cercarial exposure over time, each time point was compared to its preceding time point and Week 0. The glmQLFit() function was used to model gene expression across samples, followed by glmQLFTest() to assess differential expression. Batch and participant identifiers were included in the model to control for potential confounding effects. To assess how DEG profiles varied by experimental variables, including parasite (cercariae) sex and dose, participant sex and age, symptom grade, and schistosome circulating anodic antigen (SCAA) status, samples were stratified into subsets based on each variable. The same edgeR modeling approach was applied to each subset. Parallel analyses were thus conducted on the full dataset and all stratified subsets to identify genes associated with these variables.

**DEG Enrichment and Pathway Analyses:** To assess functional changes across comparisons, two enrichment methods were used. For comparisons with identified DEGs, Gene Ontology (GO) enrichment analysis based on the hypergeometric distribution was conducted to identify affected biological pathways. This approach highlights pathways influenced by genes with substantial expression changes. However, because the number of DEGs varied significantly across comparisons, Gene Set Enrichment Analysis (GSEA) was also performed to ensure comparability(12). GSEA considers the fold change of all genes, allowing the identification of pathways in which multiple genes change modestly but in a coordinated manner. In GSEA, all expressed genes were ranked by fold change, and gene sets derived from specific pathways were mapped onto this ranked list. Under the null hypothesis, genes from an unaffected pathway are randomly distributed; significant deviations from this distribution suggest biological relevance. Pathways enriched at the top of the list (enrichment score > 0) indicate activation, while those enriched at the bottom (enrichment score < 0) suggest suppression. Both GO enrichment and GSEA were performed using the clusterProfiler R package(13). GO enrichment used the enrichGO() function with the biological process ontology from the org.Hs.eg.db database. GSEA was conducted using the gseGO() and gseKEGG() functions, referencing GO and Kyoto Encyclopedia of Genes and Genomes (KEGG) gene sets, respectively.

To identify the most affected pathways consistently enriched across distinct subgroups, only GO/KEGG terms enriched in mutually exclusive groups (i.e., groups stratified by the same experimental variable with no overlapping participants) were considered. This approach enhances reliability and minimizes sampling bias. FDR-adjusted *P* values for these terms from each comparison were then combined using Fisher's method. Finally, the top 10 terms with the smallest combined *P* values (*P_adj.comb_*) were selected. These terms, along with their individual *P_adj_* from each comparison, were visualized in heatmap.

To detect enrichment of specific immune-related GO terms, a keyword matching approach using regular expressions was employed. Functional descriptions, including the *Synonym* and *Definition* fields for each Biological Process GO ID, were retrieved from the *org.Hs.eg.db* R package. These descriptions were searched using predefined keywords (‘Type 1 immunity’, ‘type 2 immunity’, ‘Th1’, ‘Th2’, ‘Th17’, ‘antigen’, ‘cytokine’, ‘chemokine’, ‘MHC’, ‘interferon’, ‘interleukin’, ‘inflammatory’, ‘adaptive immune’, ‘innate immune’, ‘pattern recognition receptor’, ‘T cell’, ‘B cell’, ‘lymphocyte’, ‘leukocyte’, ‘NF-KappaB’, ‘tumor necrosis factor’, ‘toll-like receptor’, ‘MAPK’, ‘natural killer’, ‘neutrophil’, ‘macrophage’, ‘humoral immune’, and ‘eosinophilic’) to identify GO terms related to corresponding immune function. Matched GO terms and their descendant terms were cross-referenced with enrichment results to extract their *P_adj_*. Then all *P_adj_* for each term were combined based on Fisher’s method to generate a single *P_adj.comb_* for each term. At last, top GO terms with smallest *P_adj.comb_* were selected and visualized in heatmaps with its original *P_adj_*.

**Fuzzy C-Means Clustering and Transcription Factor Analysis:** Fuzzy clustering identifies genes exhibiting synchronous expression changes across time points. To conduct trajectory clustering analysis, fuzzy clustering was performed using the Mfuzz R package, which assigns each gene to clusters with a gradual degree of membership ranging from 0 to 1(14). Due to noticeable individual heterogeneity, which can affect clustering results in small groups, only subgroups with at least 10 participants were included in this analysis. For each subgroup of samples, the analysis began by calculating the average log2CPM values for each gene across time points. These values, representing gene expression levels, were then scaled to ensure comparability across genes. The optimal number of clusters was estimated using the elbow method with two R functions: fviz_nbclust() from the factoextra package, which computed the within-cluster sum of squares (WCSS) across 1 to 10 clusters, and kneedle() from the kneedle package, which identified the inflection point in the WCSS curve. Genes were clustered using the mfuzz() function, with the parameter *c* set to the estimated optimal number of clusters. Genes with a membership value ≥ 0.7 were retained as core members of each cluster. To evaluate the consistency of gene clustering across sample subgroups, the adjusted Rand index was calculated using the adjustedRandIndex() function from the mclust R package.

To identify gene groups that consistently co-cluster across conditions, a second-round meta-clustering was performed based on Gower's distance for each gene, which effectively handles categorical data generated from C-means clustering. The gene groups identified through meta-clustering were further intersected with DEGs from Weeks 4 and 8. GO enrichment analysis was performed to characterize the biological functions of each gene group and their DEG intersections, using the same approach described in the DEG analysis section. Additionally, transcription factor enrichment analysis was conducted by submitting gene lists to the Enrichr server(15), using the Transcription Factor Protein-Protein Interaction database.

**Weighted Gene Co-expression Network Analysis (WGCNA):** WGCNA was performed on all samples to construct weighted gene co-expression networks and identify gene modules associated with experimental variables(16). Samples were firstly clustered using Euclidean distance, and then outlier samples were identified and excluded by setting the cutHeight parameter to 110 in the cutreeStatic() function, resulting in the retention of 111 out of 144 samples for network construction. As recommended, the soft-thresholding power parameter (β) was optimized using the pickSoftThreshold() function and set to 10. Gene co-expression networks were then constructed using the blockwiseModules() function with both TOMtype and networkType set to "unsigned", a minimum module size (minModuleSize) of 30, and a mergeCutHeight of 0.25 to merge similar modules.

**Enriched Gene Module Changes for Individual Samples:** To assess transcriptional changes at the individual participant level, we applied a blood transcriptional module repertoire analysis using BloodGen3Module, which incorporates 985 reference blood transcriptome profiles(17). For each participant, gene expression levels (log2CPM) at each post-infection time point were subtracted from baseline (Week 0) values. Genes were then ranked based on these differences, which represent log2 fold changes relative to the baseline. Gene symbols corresponding to 382 predefined functional gene sets (modules) from BloodGen3Module were extracted(17). These modules, along with the ranked gene lists, were used as input for GSEA via the fgsea R package(18), which implements the same algorithm as clusterProfiler but with greater computational efficiency. For each participant, modules with significant enrichment (*P* < 0.05) were retained and visualized as heatmaps.

Supplementary Figures and Supplementary Tables

Supplementary Figures


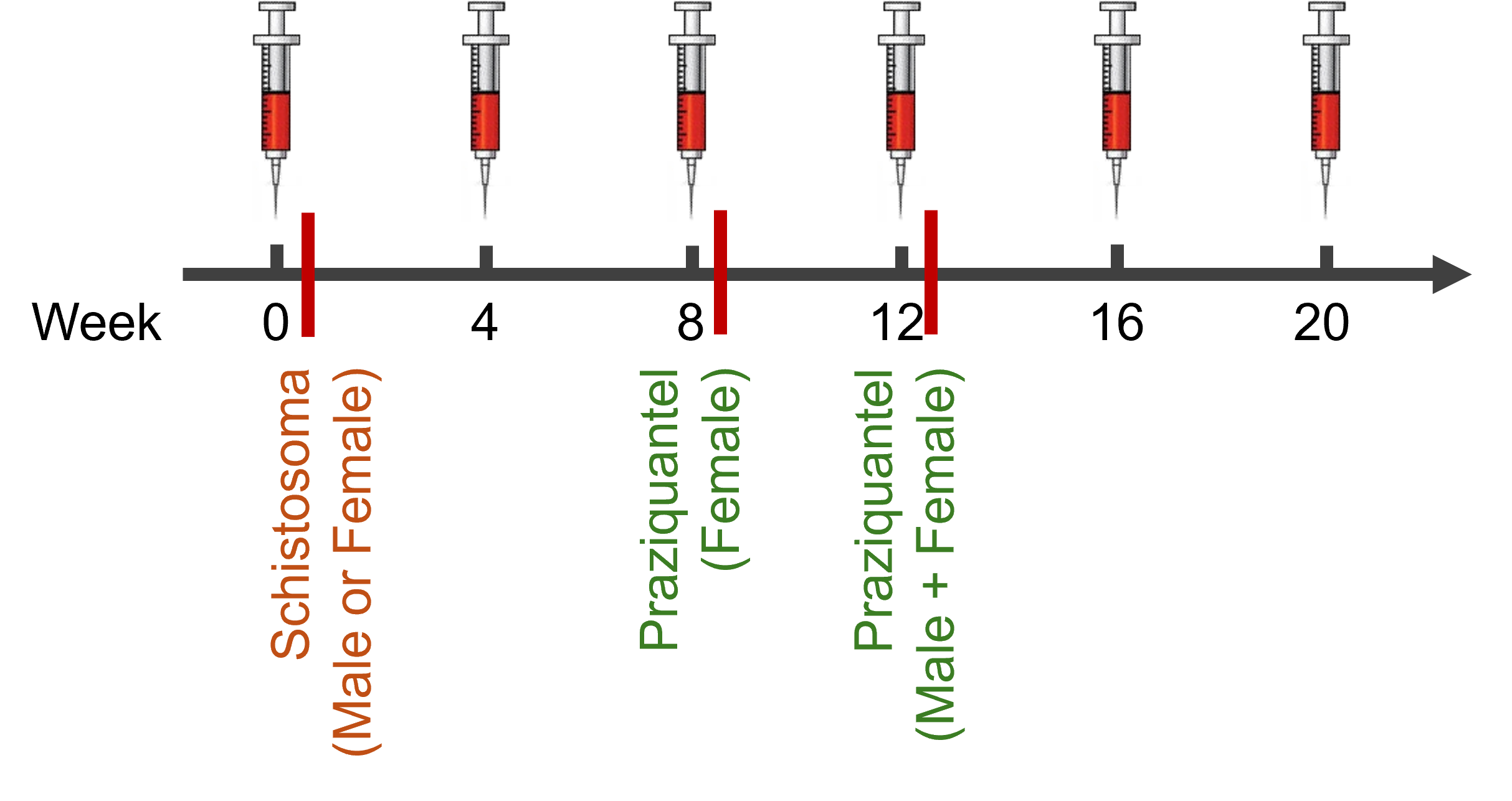


Fig. S1. Schistosoma exposure, blood collection, and praziquantel treatment.


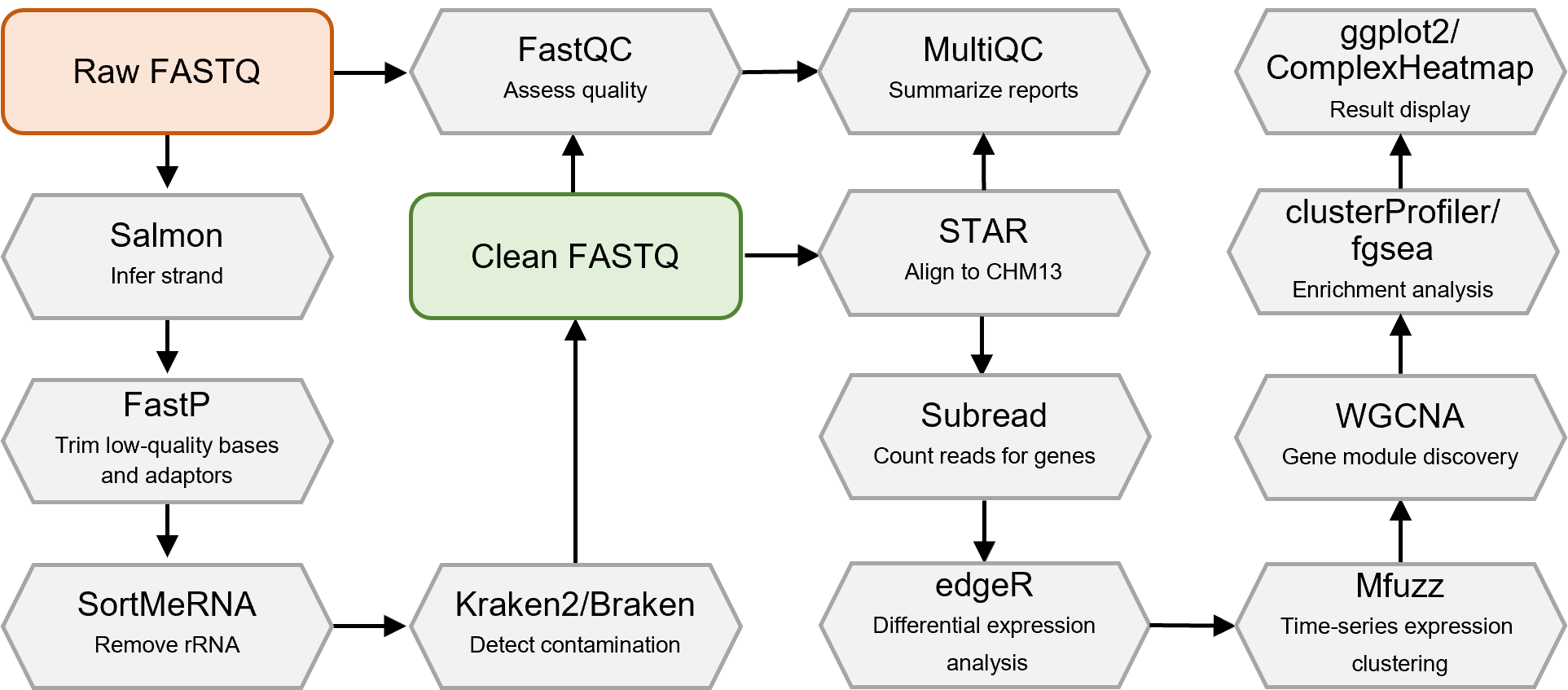


Fig. S2. Diagram of transcriptomic analysis pipeline.


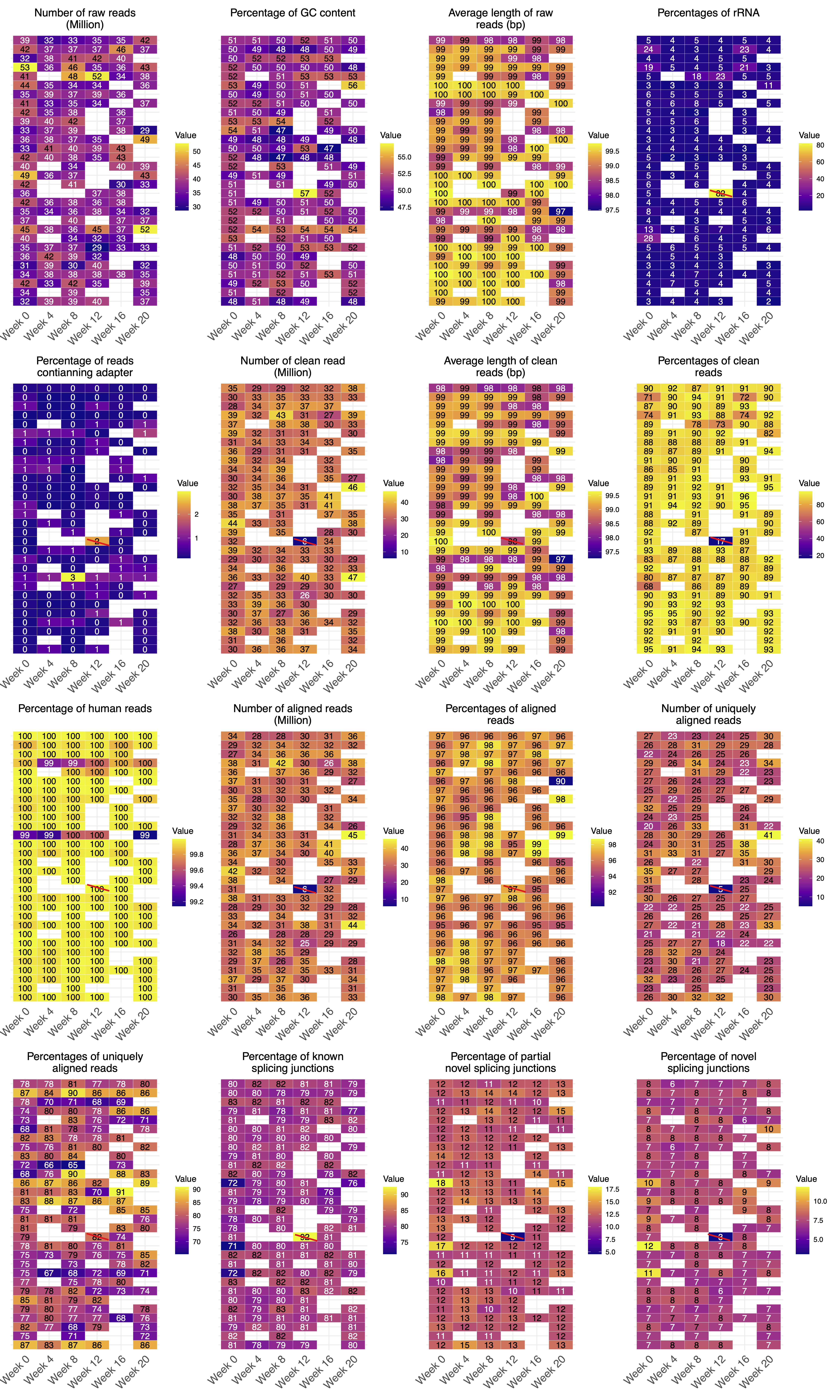


**Fig. S3.** Summary of total read counts, percentages of aligned reads, uniquely aligned reads, rRNA content, and percentage of human reads for each sample.


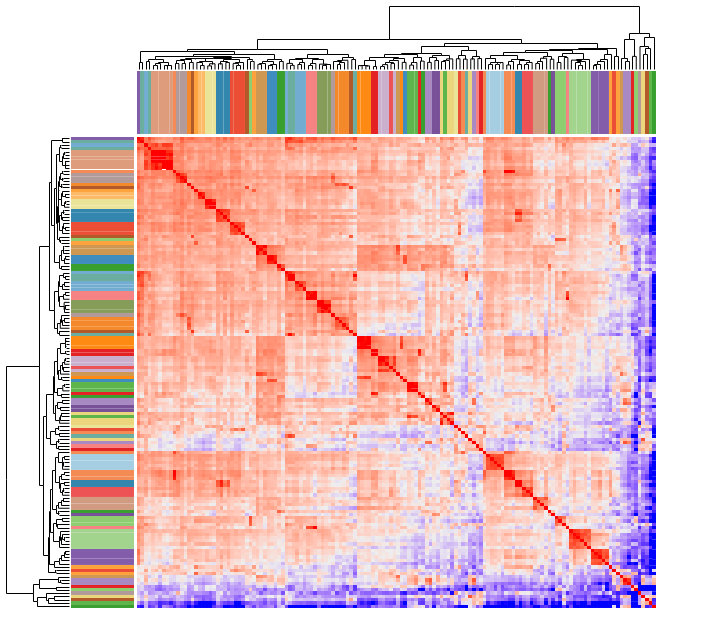


Fig. S4. Pairwise sample correlation heatmaps. Samples from the same participant are annotated using the same color along the top and side of the matrix. The heatmap displays correlation coefficients between all sample pairs.


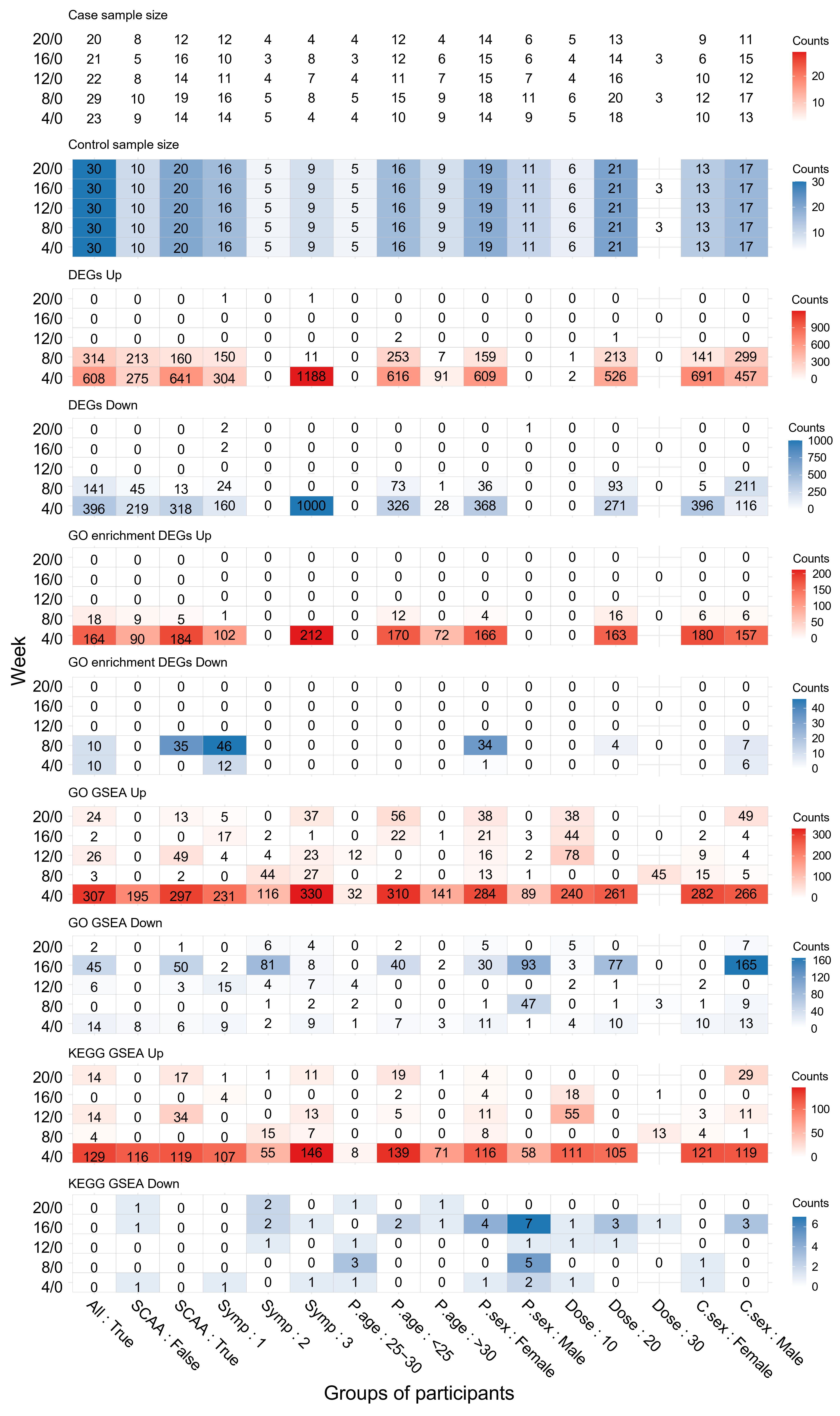


A

C

B

D

F

E

G

I

H

G

**Fig. S5.** Summary of sample sizes, number of DEGs, and number of enriched GO terms.


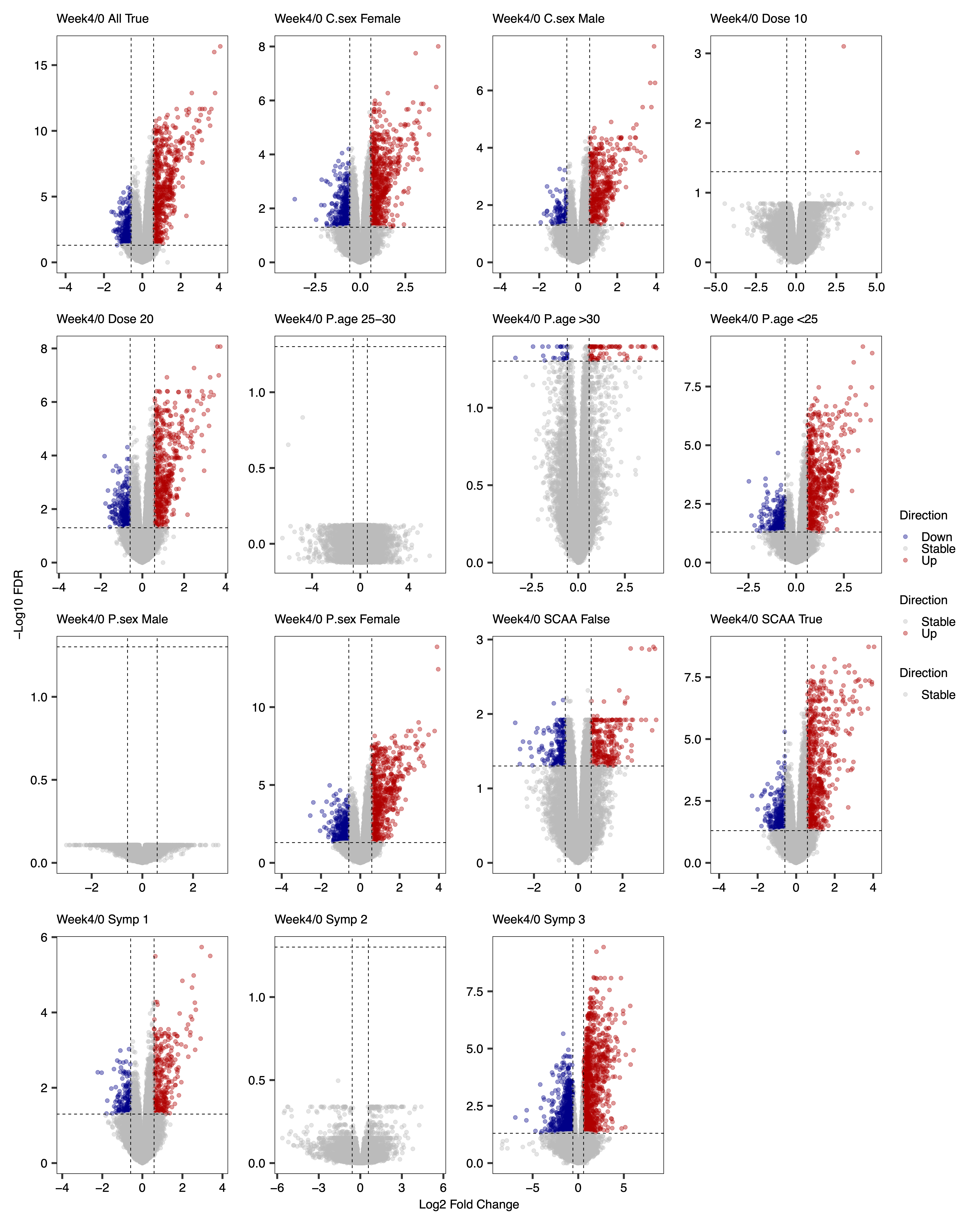


Fig. S6. Volcano plots comparing Week 4 vs. Week 0.

**
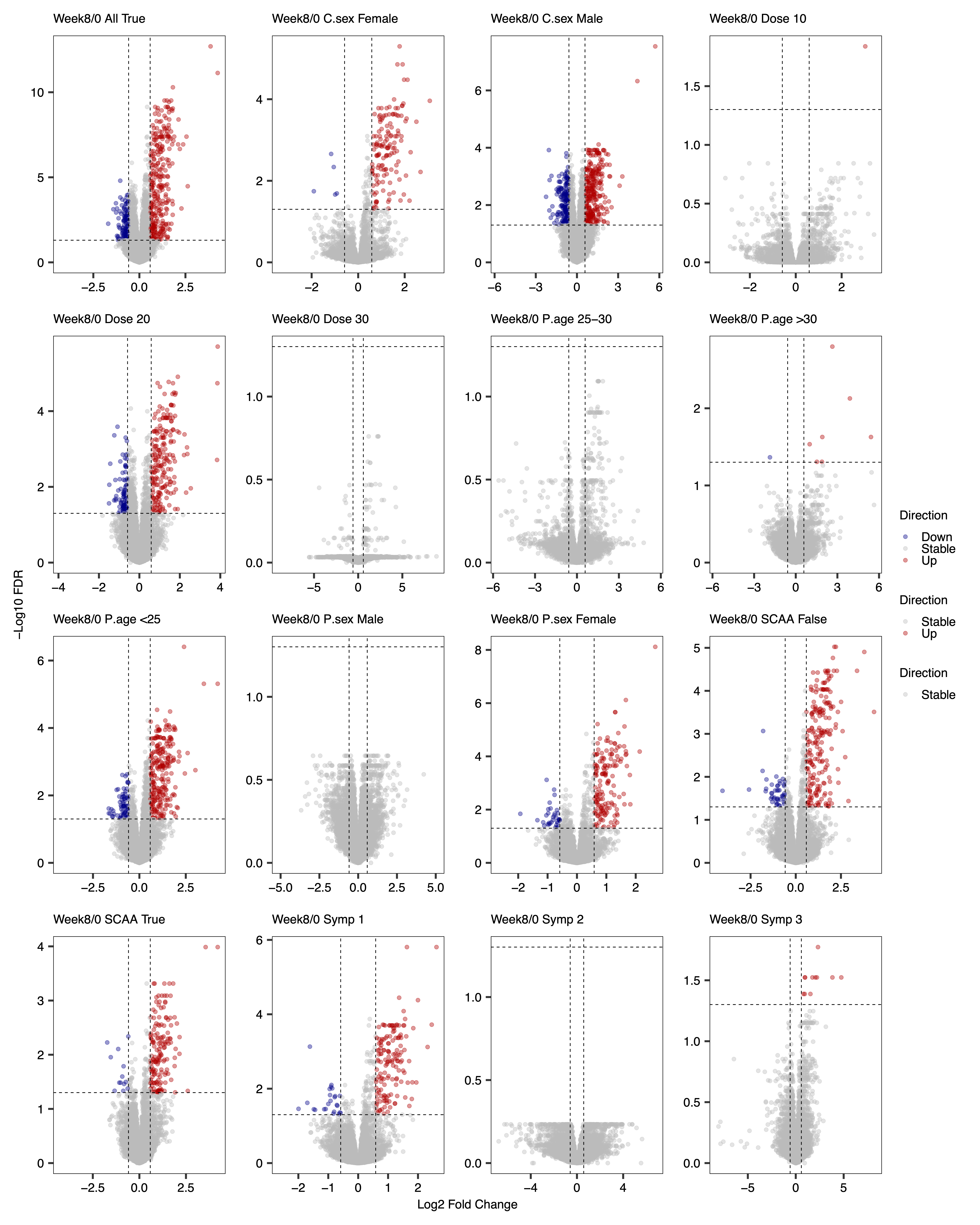
**

Fig. S7. Volcano plots comparing Week 8 vs. Week 0.


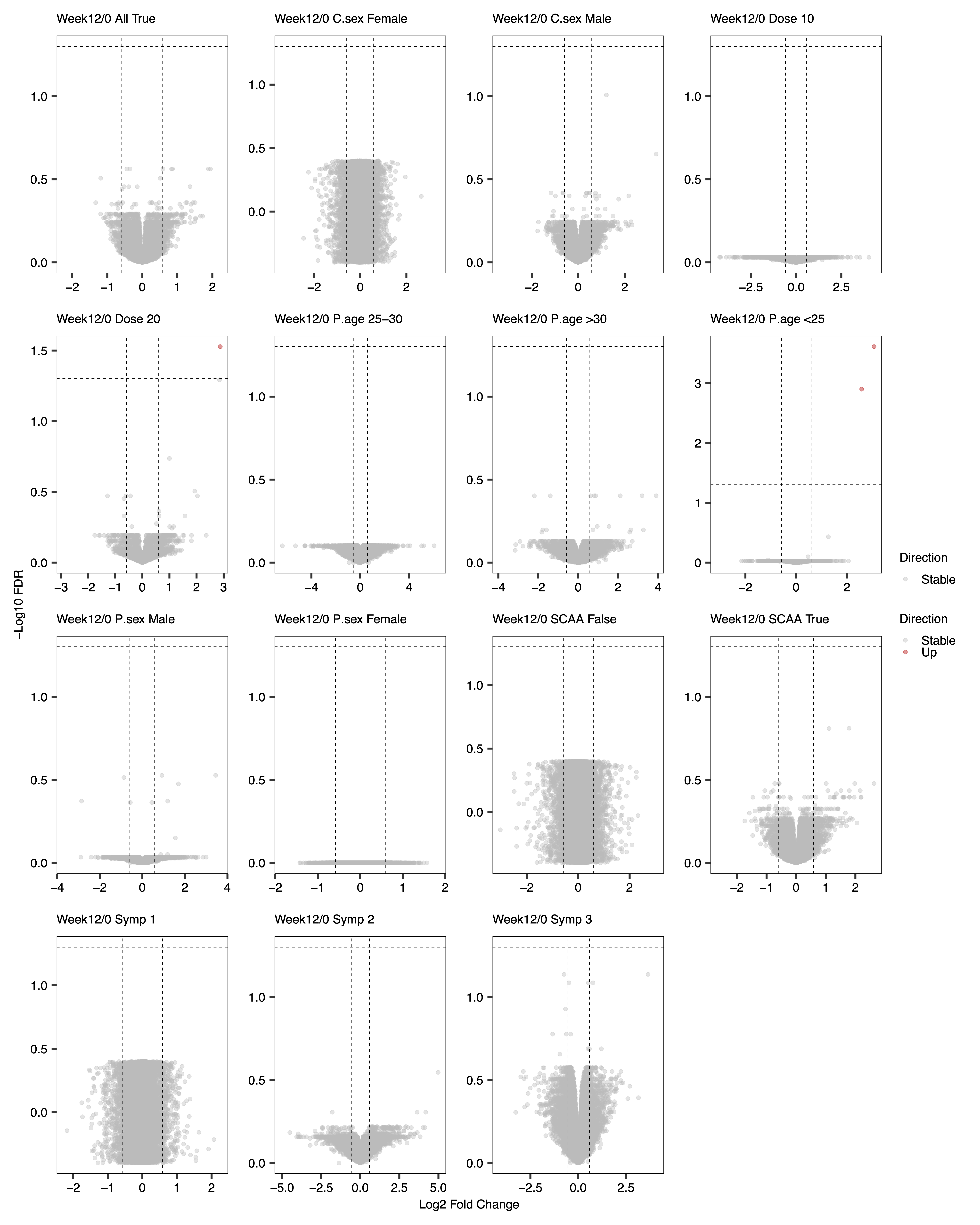


Fig. S8. Volcano plots comparing Week 12 vs. Week 0.

**
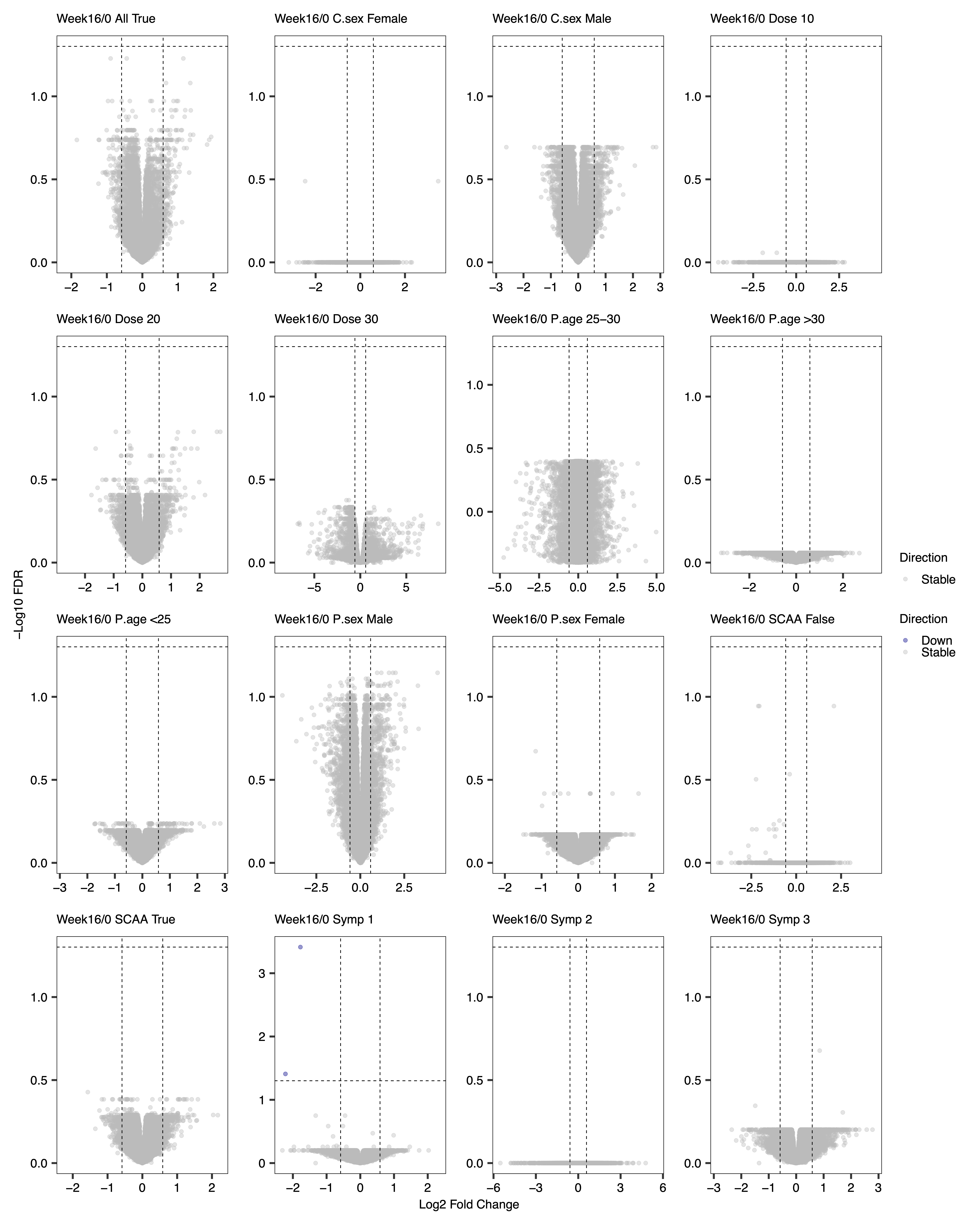
**

Fig. S9. Volcano plots comparing Week 16 vs. Week 0.

**
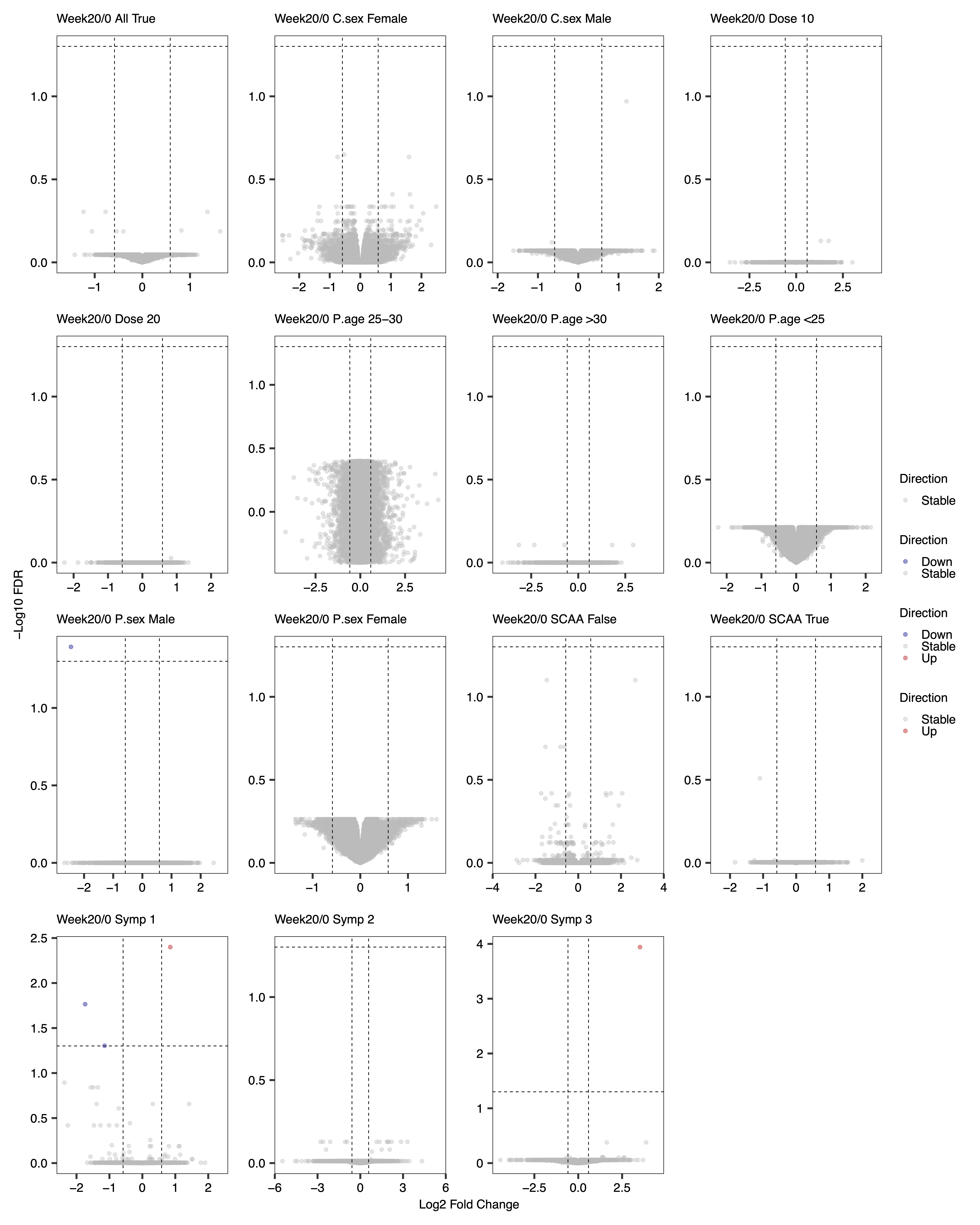
**

Fig. S10. Volcano plots comparing Week 20 vs. Week 0.


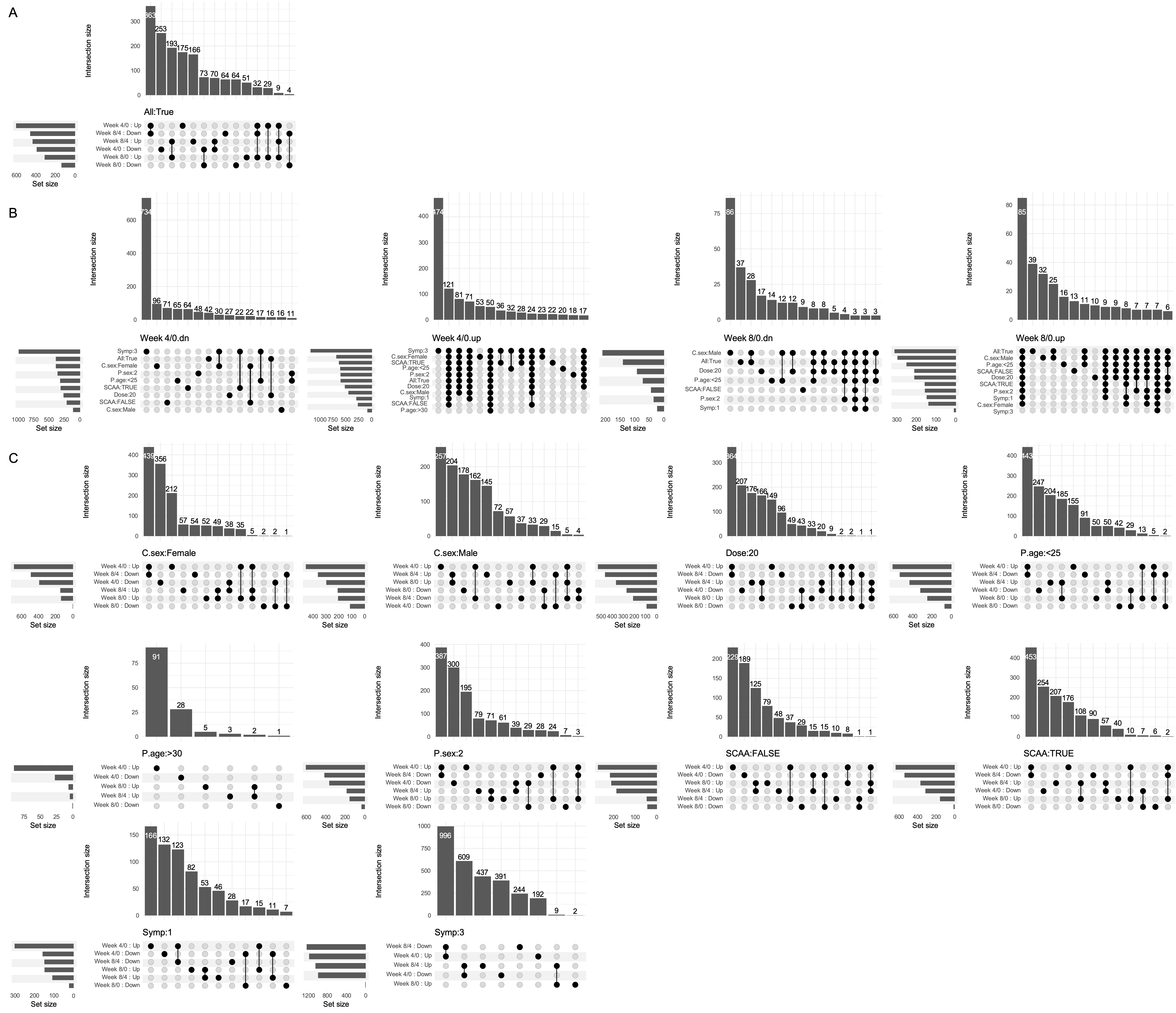


Fig. S11. Intersection of DEGs across weeks and various subgroups. (A) Intersections of DEGs identified from all samples or from participant subgroups classified based on experimental variables. (B) Four UpSet plots correspond to upregulated and downregulated DEGs at Week 4 and Week 8, respectively. (C) Intersections of DEGs identified by comparing Week 4 and Week 8 with Week 0. Each UpSet plot represents a comparison performed on all samples or on subgroups defined by experimental variables. In each UpSet plot, horizontal bars on the left indicate the total number of DEGs in each individual set. The dot matrix below the top bar chart specifies which sets are included in each intersection. Vertical bars above the matrix represent the number of DEGs shared among the selected sets. Only the top 15 intersections with the highest number of DEGs are shown.


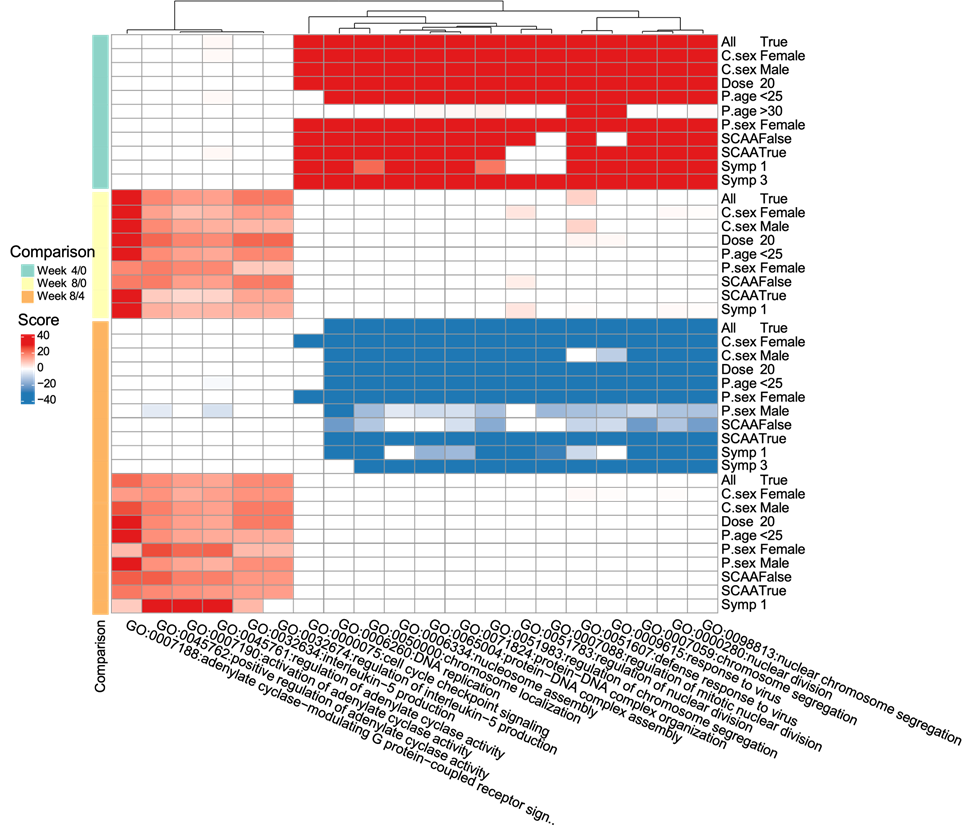


Fig. S12. GO terms repeatedly detected by enrichment analysis across time-point comparisons in subgroups. Color score reflects -log₁₀(*P_adj_*)×10. Positively and negatively enriched GO terms are shown in red and blue, respectively.


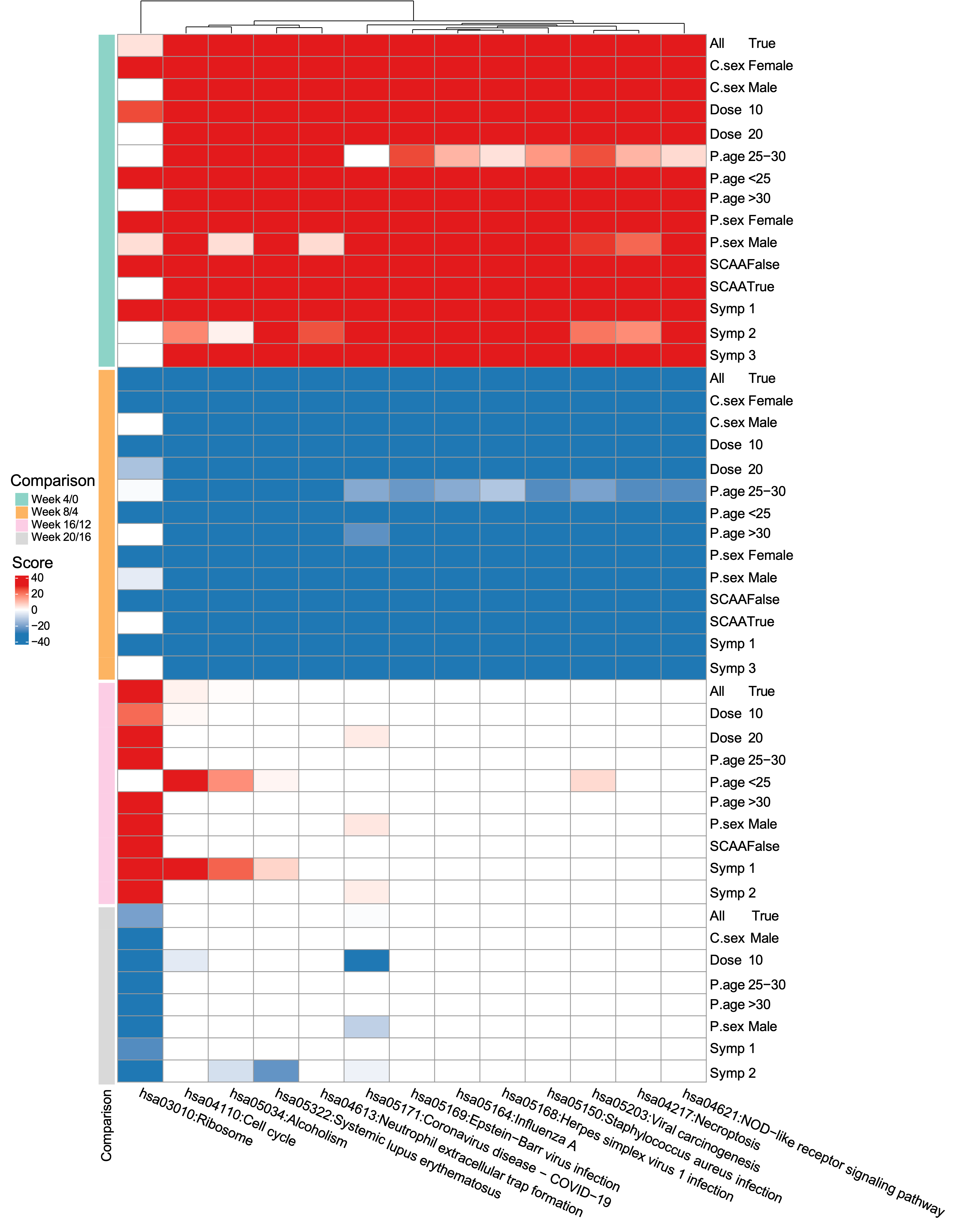


Fig. S13. KEGG terms repeatedly detected by GSEA across time-point comparisons in subgroups. Color score reflects -log₁₀(*P_adj_*)×10. Positively and negatively enriched terms are shown in red and blue, respectively.


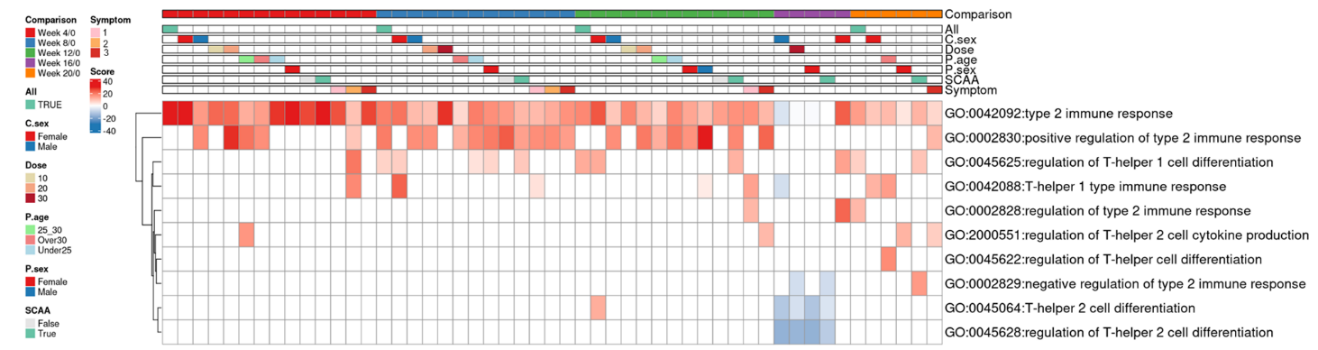


Fig. S14. GO terms related to Th1 and Th2 immune responses (cutoff *P* < 0.05). Color score reflects -log₁₀(*P*)×10. Positively and negatively enriched terms are shown in red and blue, respectively.


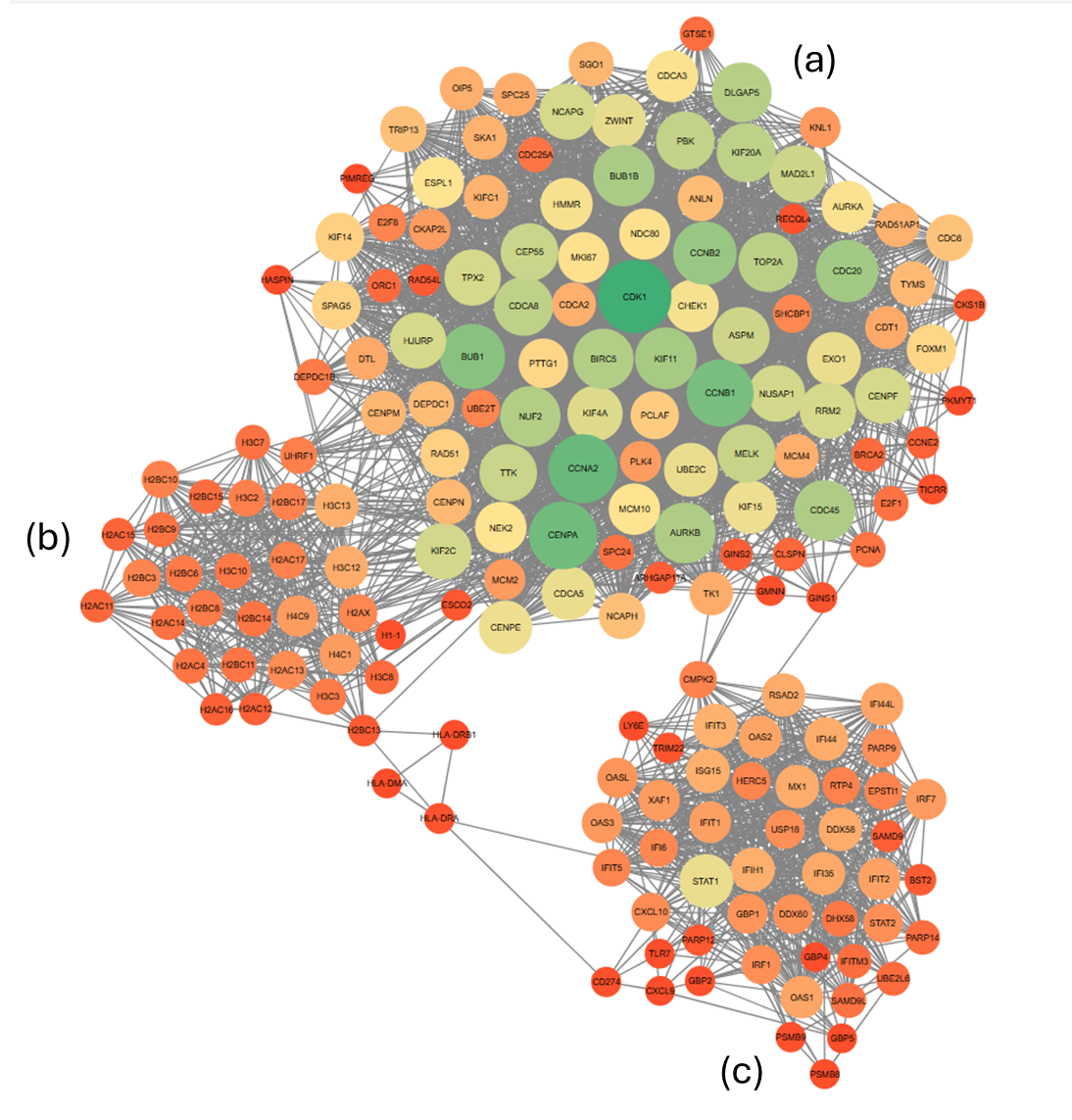


Fig. S15. PPI network constructed using upregulated DEGs at Week 4.

**
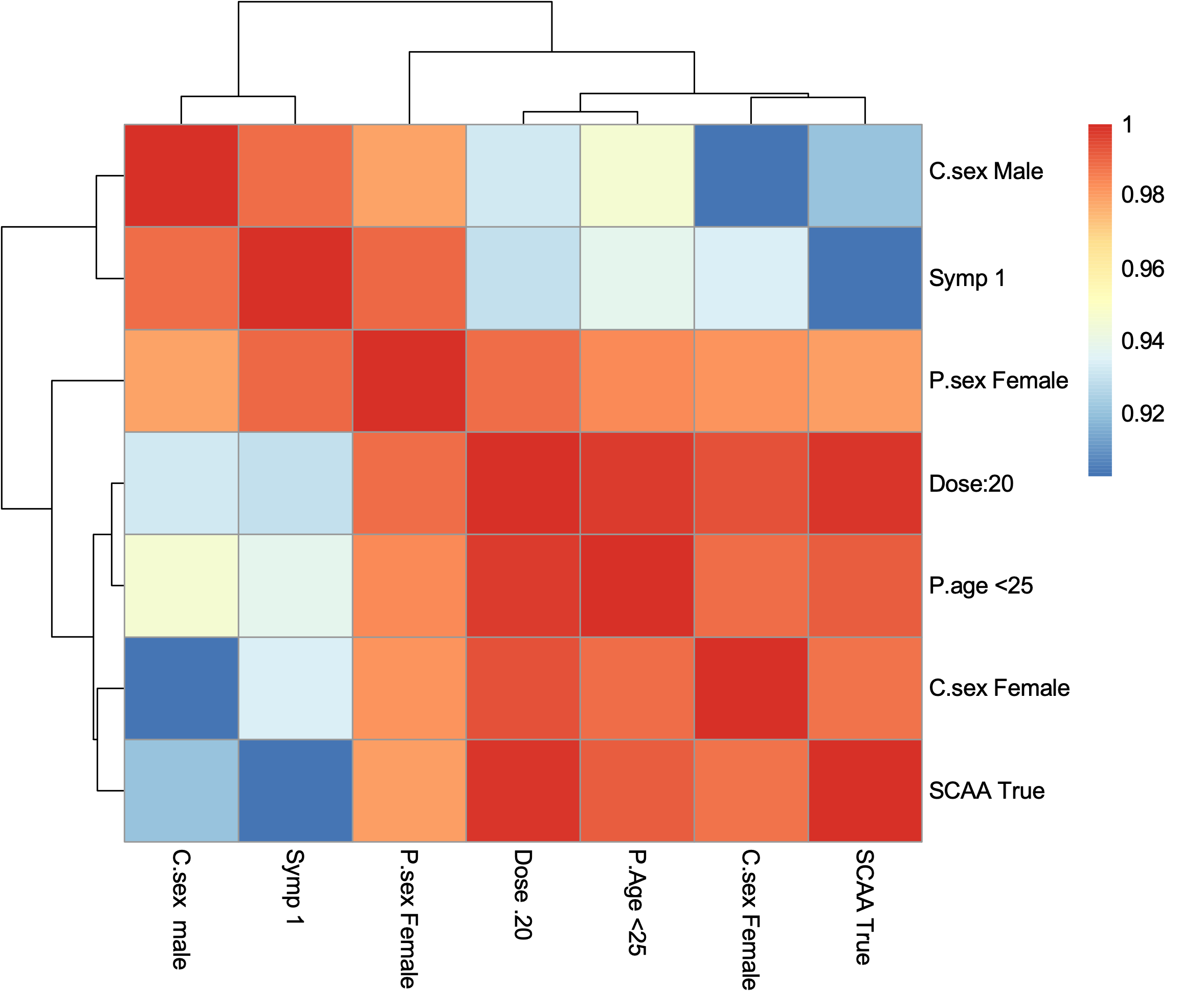
**

Fig. S16. Heatmap of ARI values for clustering agreement between sample subgroup pairs.





Fig. S17. Size of WGCNA modules and their overlap with Week 4 and Week 8 DEGs. (A) Size of WGCNA modules. (B, C) Count and percentage of Week 4 upregulated (upward bars) and downregulated (downward bars) DEGs within each module. (D, E) Count and percentage of Week 8 upregulated (upward bars) and downregulated (downward bars) DEGs within each module.

**Supplementary Tables (Excel)**

**Supplementary Table 1** Breakdown of sample sizes across all subgroups

**Supplementary Table 2** Differentially expressed genes (DEGs) across various comparisons

**Supplementary Table 3** Enriched gene ontology (GO) terms of DEGs

**Supplementary Table 4** GSEA results from GO and KEGG annotations

**Supplementary Table 5** C-Means clustering of gene expression in various subgroups

**Supplementary Table 6** Enriched GO terms in Group 1 genes: all Group 1 genes (6A) and unique Group 1 genes in comparison with Week 4 DEGs (6B)

**Supplementary Table 7** Enriched transcription factors in Group 1 genes: all Group 1 genes (7A), unique Group 1 genes in comparison with Week 4 DEGs (7B), and Group 1 genes overlapped with Week 4 DEGs (7C)

**Supplementary Table 8** Gene modules identified by WGCNA

**Supplementary Table 9** Enriched GO terms in WGCNA gene modules

**References**

1. Andrews S. FastQC: A Quality Control Tool for High Throughput Sequence Data. Available online at: <http://wwwbioinformaticsbabrahamacuk/projects/fastqc>. 2010.

2. Patro R, Duggal G, Love MI, Irizarry RA, Kingsford C. Salmon provides fast and bias-aware quantification of transcript expression. Nat Methods. 2017;14(4):417-9.

3. Wang L, Wang S, Li W. RSeQC: quality control of RNA-seq experiments. Bioinformatics. 2012;28(16):2184-5.

4. Chen S, Zhou Y, Chen Y, Gu J. fastp: an ultra-fast all-in-one FASTQ preprocessor. Bioinformatics. 2018;34(17):i884-i90.

5. Kopylova E, Noe L, Touzet H. SortMeRNA: fast and accurate filtering of ribosomal RNAs in metatranscriptomic data. Bioinformatics. 2012;28(24):3211-7.

6. Lu J, Rincon N, Wood DE, Breitwieser FP, Pockrandt C, Langmead B, et al. Metagenome analysis using the Kraken software suite. Nat Protoc. 2022;17(12):2815-39.

7. Chen X, Li D. Sequencing facility and DNA source associated patterns of virus-mappable reads in whole-genome sequencing data. Genomics. 2021;113(1 Pt 2):1189-98.

8. Ewels P, Magnusson M, Lundin S, Kaller M. MultiQC: summarize analysis results for multiple tools and samples in a single report. Bioinformatics. 2016;32(19):3047-8.

9. Dobin A, Davis CA, Schlesinger F, Drenkow J, Zaleski C, Jha S, et al. STAR: ultrafast universal RNA-seq aligner. Bioinformatics. 2012;29(1):15-21.

10. Liao Y, Smyth GK, Shi W. featureCounts: an efficient general purpose program for assigning sequence reads to genomic features. Bioinformatics. 2013;30(7):923-30.

11. Chen Y, Chen L, Lun Aaron TL, Baldoni Pedro L, Smyth Gordon K. edgeR v4: powerful differential analysis of sequencing data with expanded functionality and improved support for small counts and larger datasets. Nucleic Acids Research. 2025;53(2).

12. Subramanian A, Tamayo P, Mootha VK, Mukherjee S, Ebert BL, Gillette MA, et al. Gene set enrichment analysis: A knowledge-based approach for interpreting genome-wide expression profiles. Proceedings of the National Academy of Sciences. 2005;102(43):15545-50.

13. Yu G, Wang L-G, Han Y, He Q-Y. clusterProfiler: an R Package for Comparing Biological Themes Among Gene Clusters. OMICS: A Journal of Integrative Biology. 2012;16(5):284-7.

14. FUTSCHIK ME, CARLISLE B. NOISE-ROBUST SOFT CLUSTERING OF GENE EXPRESSION TIME-COURSE DATA. Journal of Bioinformatics and Computational Biology. 2005;03(04):965-88.

15. Xie Z, Bailey A, Kuleshov MV, Clarke DJB, Evangelista JE, Jenkins SL, et al. Gene Set Knowledge Discovery with Enrichr. Current Protocols. 2021;1(3).

16. Langfelder P, Horvath S. WGCNA: an R package for weighted correlation network analysis. BMC Bioinformatics. 2008;9(1):559.

17. Rinchai D, Roelands J, Toufiq M, Hendrickx W, Altman MC, Bedognetti D, et al. BloodGen3Module: blood transcriptional module repertoire analysis and visualization using R. Bioinformatics. 2021;37(16):2382-9.

18. Korotkevich G, Sukhov V, Budin N, Shpak B, Artyomov MN, Sergushichev A. Fast gene set enrichment analysis. 2016.
